# FAM210B activates STAT1/IRF9/IFIT3 axis by upregulating IFN-α/β expression to impede the progression of lung adenocarcinoma

**DOI:** 10.1038/s41419-025-07375-9

**Published:** 2025-02-03

**Authors:** Xuejuan Gao, Donglan Huang, Ying Liu, Gui Zhang, Xiaofen Zheng, Baiye Guan, Aiwen Chen, Jiayao Wu, Shi-Ming Luo, Zonghua Liu, Luxuan Chen, Xiaohui Liu, Jingjie Jin, Xingfeng Yin, Zhenghua Sun, Yunfang Zhang, Meizhi Lu, Gong Zhang, Wanting Liu, Langxia Liu

**Affiliations:** 1 MOE Key Laboratory of Tumor Molecular Biology and State Key Laboratory of Bioactive Molecules and Druggability Assessment, Institute of Life and Health Engineering, College of Life Science and Technology, Jinan University, Guangzhou, China; 2 Guangzhou Key Laboratory of Metabolic Diseases and Reproductive Health, Guangdong-Hong Kong Metabolism & Reproduction Joint Laboratory, Reproductive Medicine Center, Guangdong Second Provincial General Hospital, Guangzhou, Guangdong China; 3 Department of Biomedical Engineering, College of Life Science and Technology, Jinan University, Guangzhou, China; 4 Department of Nephrology, People’s Hospital of Huadu District; The Third School of Clinical Medicine, Southern Medical University, Guangzhou, China

**Keywords:** Non-small-cell lung cancer, Tumour-suppressor proteins

## Abstract

FAM210B (family with sequence similarity 210 member B) is a novel protein that has been linked to tumor development. However, its role and underlying mechanisms in lung adenocarcinoma (LUAD) progression remain largely unexplored. In this study, FAM210B was observed to be down-regulated in LUAD cells. Analyses of public datasets revealed that decreased expression of FAM210B predicts poor survival. Accordingly, in vitro and in vivo studies have confirmed the inhibitory role of FAM210B on the growth and tumor metastasis of LUAD cells. RNA-seq analysis further indicated that FAM210B plays a role in regulating innate immune-related signaling pathways in LUAD cells, particularly involving the production of type I interferon (IFN-α/β). Specifically, FAM210B activates STAT1/IRF9/IFIT3 axis by upregulating IFN-α/β expression, leading to the inhibition of proliferation and migration of LUAD cells. Furthermore, TOM70 (Translocase of outer mitochondrial membrane 70, also named as TOMM70) has been identified as a functional interacting partner of FAM210B in its modulation on the expression of IFN-α/β, as well as the proliferative and metastatic phenotypes of LUAD cells. In conclusion, our study indicates that FAM210B is an important suppressor of cellular viability and mobility during lung cancer progression.

## Introduction

Lung cancer is a highly malignant form of cancer with high morbidity and fatality rates, making it a major global health concern [1, 2]. There are two main subtypes of lung cancer: non-small cell lung cancer (NSCLC) and small cell lung cancer (SCLC). NSCLC accounts for 85% of lung cancers, mainly comprised of lung adenocarcinoma (LUAD, 40%) and lung squamous cell carcinoma [3]. Despite advancements being made in tumor therapeutic strategies in the recent years, the five-year survival rate for individuals suffering from lung cancer remains poor [3]. Further understanding of LUAD pathogenesis is critical for the development of new therapy for this devastating illness.

Type I interferons (IFN) are major immune response regulators mainly consist of several IFN-α subtypes and one IFN-β subtype, with otherwise the less-reported IFN-ɛ, −κ, −τ, and −ω subtypes. These proteins exert their functions through binding to a heterodimer receptor of IFNAR1/IFNAR2. The ligand-receptor complex recruits and phosphorylates the signal transducers and activators of transcription 1 (STAT1) and STAT2, which then interact with interferon regulatory factor 9 (IRF9), and is translocated to the nucleus to activate the transcription of interferon-stimulated genes (ISGs) [4, 5]. Both the paracrine and autocrine signaling of IFN have been reported to play important roles in tumor development, respectively, by regulating the anti-tumor immune response or the cellular processes and functions of the tumor cells [6–11].

Taking part in the Chromosome-centric Human Proteome Project (C-HPP), our team has been recently dedicated to studying the role of newly identified genes on Chromosome 20 in tumor development, as demonstrated by our previous research on ZSWIM1’s involvement in the pathogenesis of LUAD [12] and the role of C20orf24 in promoting colorectal cancer progression [13]. *FAM210B* (family with sequence similarity 210 member B, C20orf108) is another newly identified gene located on Chromosome 20 and is widely expressed in human tissues [14]. Based on its significant regulatory effect on LUAD cell proliferation in a preliminary screening assay, we have selected this gene for further studies aiming to elucidate its functions in relation to LUAD cancer development. Up to date, little has been known about its physiological function, and even less about the underlying molecular mechanisms. In the context of tumor development, downregulation of FAM210B has been linked to decreased survival rates for tumor patients and increased growth and metastasis of tumor cells [15, 16]. In ovarian cancer, the loss of FAM210B was found to reduce the expression of pyruvate dehydrogenase kinase 4 (PDK4) and glycolysis, promoting epithelial-to-mesenchymal transition (EMT), migration, and invasion [15]. In hepatocellular carcinoma (HCC), knockdown of FAM210B protein was associated with increased activation of MAPK signaling and AKT signaling pathways [16]. Moreover, altered expression of FAM210B in peripheral blood has been identified as part of a 16-gene signature for predicting immune-related adverse events of severe diarrhea during anti-CTLA-4 immunotherapy [17]. FAM210B was found to be associated with mammary tumor incidence in rats [18]. These previous studies suggested a possible association of this gene with cancer. Further in-depth exploration in the field appears meaningful.

In this study, we demonstrate the significant inhibitory role of FAM210B in LUAD growth and metastasis and explore the related molecular mechanism. By using RNA-seq assay and interactome analysis, we show that FAM210B activates the innate immune pathway in LUAD cells by interacting with TOM70 (Translocase of outer mitochondrial membrane 70, also named TOMM70), leading to the production of type I interferon and ultimately inhibiting the growth and metastasis of LUAD cells. Our data provide novel insights into the pathogenesis of LUAD and identify FAM210B as a potential therapy target for this disease.

## Materials and methods

### Cell lines and culture

Human bronchial epithelial (HBE) cells and human non-small cell lung adenocarcinoma cell lines H1299 and A549 were obtained from Cell Resource Center, Institute of Life Science Chinese Academy of Sciences (Shanghai, China) and cultured in DMEM (Gibco BRL, USA) and supported with 10% FBS (Procell Life Science & Technology Co., Ltd, Wuhan, China) in a humidified chamber at 37 °C with 5% CO_2_ as described previously [12].

### Overall survival analysis

Kaplan-Meier (KM) Plotter (https://kmplot.com/analysis/) was utilized to analyze overall survival from the TCGA-LUAD dataset (*n* = 504) and two GEO datasets, namely GSE31210 (*n* = 226) and GSE30219 (*n* = 85). The TCGA-LUAD dataset comprises a total of 513 LUAD cases; however, 9 cases were excluded from the overall survival analysis due to missing status information, resulting in a final analysis of 504 patients. The GSE30219 dataset includes a total of 293 lung tumor patients, of which 85 cases were classified as adenocarcinoma (ADC) based on histological classification. Since our study primarily focuses on the adenocarcinoma subtype, we selected these 85 cases for survival curve analysis. Hazard ratios (HRs) with 95% confidence intervals and log-rank *P* values were calculated to assess the prognostic significance of FAM210B expression.

### Plasmid construction

The purchased pEGFP-N1-FAM210B plasmid (iGEbio Co., Ltd, Guangzhou, China) was used as template to amplify the FAM210B coding sequence (CDS) of 591 bp. The pCMV-FLAG-based plasmid FLAG-FAM210B was then constructed using seamless cloning technology (Vazyme Biotech Co., Ltd, Nanjing, China).

### Establishment of stable cells

Cells stably expressing FLAG-FAM210B were established using methods similar to those used in previous studies [12]. Briefly, recombinant FLAG-FAM210B cDNA was constructed and then inserted into the lentiviral vector PLVX-IRES-EGFP-PuroR. This recombinant plasmid, in conjunction with packaging plasmids psPAX2 and pMD2.G, was employed to generate lentiviruses in HEK-293T cells carrying PLVX-EGFP-FLAG-FAM210B. After 48 h, the culture medium carrying the lentiviruses was collected and used to infect H1299 cells, A549 cells, and luciferase-expressing A549 cells, resulting in the generation of stable cells (OE-FAM210B and OE-V, or OE-FAM210B-Luc or OE-V-Luc).

### Real-time quantitative reverse transcription PCR (qRT-PCR) assays

Total RNA extraction and cDNA quantification were performed using standard methods. The gene-specific primer sequences used in this study are provided in Table S1.

### Small interfering RNA transfection

The small interfering RNA (siRNA) sequences designed in this study are provided in Table S2. Cells were transfected with siRNA using Lipofectamine^@^2000 (Invitrogen, CA, USA) according to the manufacturer’s instructions.

### Colony formation assay

1 × 10^3^ cells/well were seeded into 6-well plates and cultured for 10–15 days. The resulting colonies were fixed with 4% paraformaldehyde for 30 min, stained with crystal violet for 15 min, and counted by ImageJ software.

### Transwell assay

The Transwell assay was performed following the protocol described previously [12]. Migration assays were conducted using Transwell chambers with polycarbonate membranes (24 wells, 8 μm pore size), while invasion assays utilized Matrigel-coated chambers (24 wells, 8 μm pore size). H1299 or A549 cells (8–10 × 10^4^/well) were seeded into the upper chamber containing serum-free DMEM. After an incubation period of 8–10 h, cells that migrated to the basal side of the membrane were fixed and stained for subsequent microscopic evaluation using an Olympus microscope (Tokyo, Japan).

### Wound healing assay

A549 cells of OE-Vector and OE-FAM210B or H1299 cells transfected with GFP-Vector or GFP-FAM210B were cultured in 6 cm petri dishes until reaching confluency. A linear wound was created by scratching, and the petri dishes were then placed on the stage of an inverted microscope equipped with phase-contrast optics to capture sequential images of the cells at 5-min intervals over a period of 24 h to observe the process of wound healing. The recording process was controlled using Cyto-MINI software (Guangzhou Shipu Photoelectric Technology Co. LTD, Guangzhou, China) as described previously [12].

### MTT assay

1 × 10^3^ cells/well were seeded into 96-well plates and cultured for the indicated durations. Then, 200 μl of MTT working solution (0.5 mg/ml) was added to each well and incubated for 4 h. After that, 150 μl of dimethyl sulfoxide was added and incubated for 10 min. The absorbance at 570 nm was measured to assess cell proliferation at each time point.

### Western blotting assay

The Western blotting assay was conducted following previously described methods [12, 19, 20]. Antibodies used in this study are as following: β-actin (#66009-1-Ig), LaminA/C (#10298-1-AP), GAPDH (#10494-1-AP), STAT1 (#10144-2-AP), IRF9 (#14167-1-AP), TOM70 (#14528-1-AP), and Vimentin (#10366-1-AP) from Proteintech (Wuhan, China); E-cadherin (#3195) from Cell Signaling Technology (Massachusetts, USA). Other antibodies used in this study were p-STAT1 (#AF3300, Affinity Biosciences, OH, USA), anti-p-IRF3 (#AF3438, Affinity Biosciences, OH, USA), IFN-α (#DF6086, Affinity Biosciences, OH, USA), IRF3 (#a19717, ABclonal, China), IFN-β (#R381675, Zenbio, USA), FLAG (#M185-3, MBL, Tokyo, Japan), and FAM210B (#NBP2-14523, NOVUS, CO, USA).

### Nuclear-cytoplasmic separation assays

H1299 cells transfected with either GFP-FAM210B or GFP-Vector were utilized to conduct nuclear-cytoplasmic separation assays. The Nuclear-Cytosol Extraction Kit (#P1200, Beijing Pulilai Gene Technology Co., Ltd, China) was employed to separate nuclear and cytoplasmic fractions according to the manufacturer’s instructions. Following this separation, western blotting was performed to analyze protein expression levels in the different fractions. The blots were probed with specific antibodies against IRF3 (#a19717, ABclonal, China) and phosphorylated IRF3 (#AF3438, Affinity Biosciences, OH, USA) to assess the localization and expression of these proteins.

### RNA-seq assay

H1299 cells were transfected with GFP-FAM210B or FAM210B siRNA for RNA-seq analysis. Total RNA was extracted using TRIzol reagent (Invitrogen, USA). Equal amounts of total RNA from three independent experiments were combined to create a library for RNA-seq, following the standard protocol provided by BGI (BGI-Shenzhen, Shenzhen, China). The library was prepared using the BGISEQ-500RS High-throughput sequencing kit (PE100, V3.0, MGI Tech Co., Ltd), as previously described [21, 22]. Differential gene expression analysis was conducted using the edgeR package, considering a *P* value threshold of less than 0.01. To perform Gene Ontology (GO) analysis on these differentially expressed genes (DEGs), the Database for Annotation, Visualization, and Integrated Discovery (DAVID) platform was utilized.

### Co-immunoprecipitation (Co-IP) assay

The cell samples were lysed in EBC buffer supplemented with a proteasome inhibitor cocktail (CWbio Co., Ltd, Taizhou, China), as well as NaF, Na_3_VO_4,_ and PMSF [12]. 1 mg of cell lysate was incubated with 2 μg of primary antibody overnight at 4 °C. Protein A/G PLUS-Agarose (Santa Cruz Biotechnology, Texas, USA) was then added to the mixture and incubated for 4–6 h. The immune complexes were subsequently subjected to five washes with EBC buffer and separated using western blotting.

### Immunofluorescence (IF) assay

H1299 cells were transfected either with pGFP-FAM210B and FLAG-TOM70 (for the colocalization of FAM210B and TOM70 proteins), or only with pGFP-FAM210B (for the localization of p-IRF3). After fixation, the cells were incubated with anti-TOM70 (#14528-1-AP, 1:200) or anti-p-IRF3 (#AF3438, Affinity Biosciences, OH, USA) overnight at 4 °C. The cells were then incubated with Alexa Fluor 594-conjugated secondary antibody (ZSGBBIO Co., Ltd, Beijing, China), and the nuclei were stained with DAPI. To observe the colocalization of GFP-FAM210B and mitochondria in live cells, H1299 cells were transfected with pGFP-FAM210B for 48 h and then stained with MitoTracker Red (100 nM, Thermo Fisher Scientific) for 20 min. These samples were observed and imaged using a confocal microscope (LSM 900, Carl Zeiss).

### Mass spectrometry analysis

H1299 cells stably expressing FAM210B (OE-FLAG-FAM210B) were lysed with lysis buffer. The lysates were then subjected to standard co-IP with anti-FLAG antibody, while non-immune IgG was used as the control group. The immunoprecipitated complexes were separated by SDS-PAGE and visualized through silver staining. The proteins in the gel were subsequently extracted using an enzymatic hydrolysis method similar to that described previously [12, 23]. The resulting peptide samples were analyzed on an Orbitrap Fusion Lumos mass spectrometer (Thermo Fisher Scientific, MA, USA). The raw data obtained from the mass spectrometer were automatically analyzed by the Sequest HT engine of the Proteome Discoverer (PD, Thermo Fisher Scientific) v2.1.1.21 against UniProtKB/Swiss-Prot *Homo sapiens* protein database with the default settings.

### Ethics statement

All animal-related procedures in this study were approved by the Experimental Animal Ethics Committee of Jinan University (Approval No: IACUC-20220219-04).

### In vivo animal experiments

The mice were purchased from Beijing Weitong Lihua Laboratory Animal Co., LTD (Beijing, China), and were raised under SPF standard conditions. For the xenograft experiment, A549 cells or H1299 cells stably overexpressing FLAG-FAM210B (OE-FAM210B) and their respective control cells (1.5–2.0 × 10^6^ cells/100 μL/mouse, *n* = 6, based on random allocation) were injected into the subcutaneous area of the armpit of BALB/c nude mice following previously described methods [24]. The tumor growth rate was monitored every 2 days, and the volume of tumor was calculated using the formula: *V* = (length x width^2^)/2. At 30–40 days of subcutaneous inoculation, the mice were euthanized by cervical dislocation, and the tumors were collected for western blot and histological analysis. No blinding was done.

For the in vivo metastasis experiment, each female NCG mouse (NOD-Prkdc^em26Cd52^Il2rg^em26Cd22^, aged 4–6 weeks, Beijing Weitong Lihua Laboratory Animal Co., LTD, Beijing, China) received an injection of luciferase-expressing OE-FAM210B-Luc or OE-V-Luc A549 cells (2 × 10^6^ cells/100 μL PBS/mouse, *n* = 6, based on random allocation) via the tail vein. After 40 days post-injection, bioluminescent imaging was performed using a Xenogen IVIS Lumina II system (PerkinElmer, MA, USA) to visualize the presence of lung metastases. Additionally, lung tissue sections were subjected to Hematoxylin and Eosin (H&E) staining to detect tumor metastasis in the lungs.

### Statistical analysis

The mean ± SEM values from three independent experiments were presented in this study. Data visualization and statistical analysis were performed using GraphPad Prism 6 software. Statistical significance was determined by performing Student’s *t*-test, or two-way ANOVA, with *P* < 0.05 considered as significant.

## Results

### FAM210B’s expression is positively associated with the prognosis of LUAD patients

**Fig. 1:**
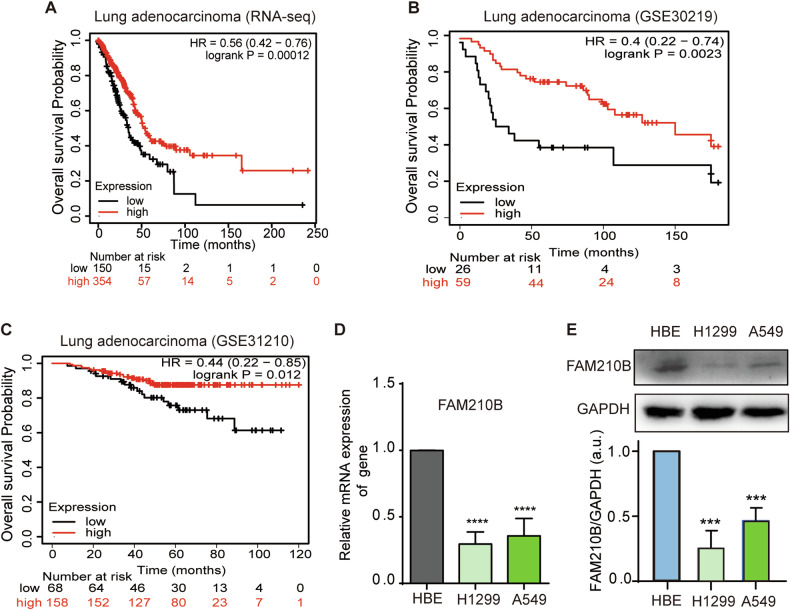
FAM210B expression level was positively correlated with overall survival in LUAD patients. Kaplan-Meier survival analysis of the relationship between FAM210B expression and overall survival in TCGA-LUAD RNA-seq data (**A**), GSE30219 (**B**), and GSE31210 (**C**), with high expression of FAM210B denoted in red and low expression of FAM210B in black. **D** qRT-PCR was used to analyze the expression levels of *FAM210B* gene in HBE, H1299, and A549 cell lines. **E** Western blot analysis of FAM210B protein expression in HBE, H1299, and A549 cell lines (Upper). Quantitative analysis of FAM210B protein expression of three independent repeated experiments (Lower). Data of D-E were represented as mean ± SEM of three independent experiments. Statistical analysis was performed using Student’s *t*-test. ***P < 0.001, ****P < 0.0001.

We investigated the correlation between FAM210B expression and overall survival in patients with LUAD. Kaplan-Meier analysis showed a statistically significant link between high FAM210B expression and improved overall survival among LUAD patients (Fig. 1A–C), suggesting a positive correlation between FAM210B expression and the prognosis of LUAD. Furthermore, FAM210B mRNA and protein levels in A549 and H1299 LUAD cells were significantly lower than that of normal HBE cells (Fig. 1D–E), hinting FAM210B might be involved in the progression of LUAD. However, we did not observe differential expression of the *FAM210B* gene in normal and tumor tissues of LUAD patients when analyzing TCGA data (Fig. S1A). In evaluating the expression of FAM210B across different stages of LUAD, specifically the terminal respiratory unit (TRU), proximal-inflammatory (PI), and proximal-proliferative (PP) subtypes, as referenced in Collisson et al.‘s study [25], the results shown in Fig. S1B indicated that tumor samples in the TRU stage (which harbored the majority of EGFR-mutated tumors) exhibited a slight increase in expression compared to the PP and PI stages. However, the implications of this increase require further investigation.

### FAM210B suppressed LUAD cell proliferation in vitro and LUAD tumor growth in vivo

**Fig. 2:**
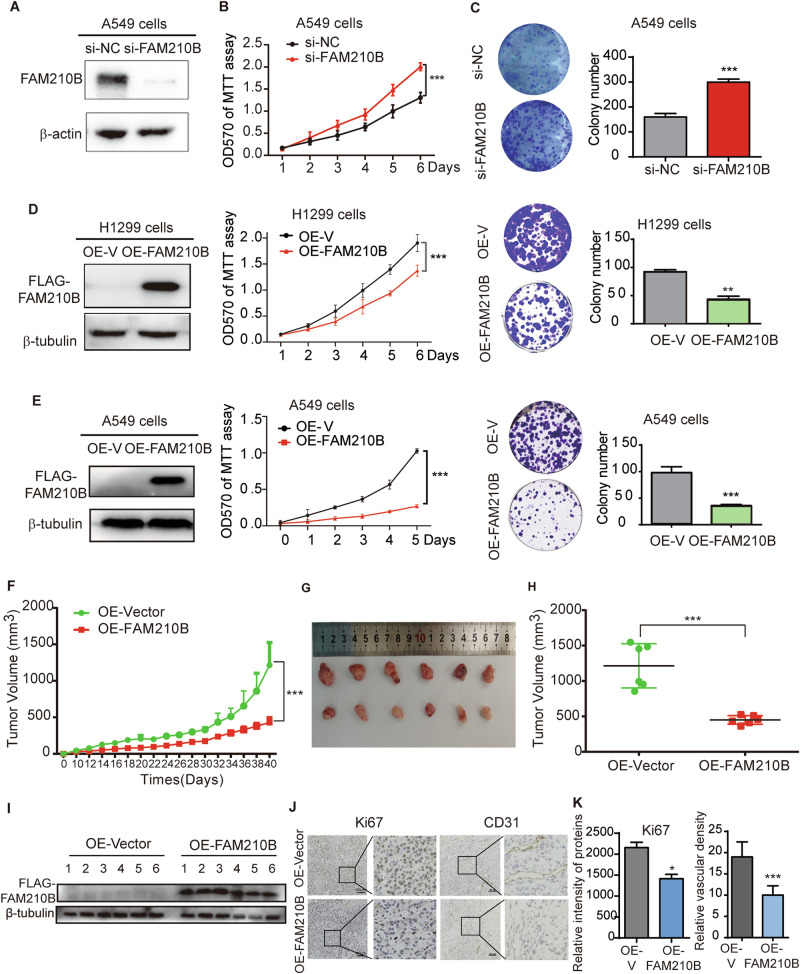
FAM210B suppressed LUAD cell proliferation in vitro and LUAD tumor growth in vivo*.* **A** Western blot analysis of FAM210B protein expression after its knockdown in A549 cells. **B** MTT assay of A549 cells after FAM210B knockdown. **C** Colony formation assays of A549 cells after FAM210B knockdown. **D** H1299 cells stably overexpressing FAM210B (OE-FAM210B, left panel) were subjected to MTT (middle panel) and colony formation assays (right panel). **E** A549 cells stably overexpressing FAM210B (OE-FAM210B, left panel) were subjected to MTT (middle panel) and colony formation assays (right panel). **F**–**H** FAM210B inhibited LUAD tumor growth in vivo. Assays were performed with the injection of either OE-FAM210B A549 cells or the control OE-Vector A549 cells. The results presented for the indicated nude mouse groups are: evolution of tumor volume measured at different time points (**F**, 40 days after subcutaneous injection of A549 OE-V and OE-FAM210B cells, the tumor volume neared the upper limit allowed by animal ethics guidelines, prompting the termination of the experiment), images of tumors at the endpoints of time (**G**), and the statistical representation of tumor volumes illustrated in (**G**, **H**). **I** Western blot assays were performed on the tumor tissues from the indicated nude mouse groups to confirm the overexpression of FAM210B. **J** IHC analyses of Ki67 and CD31 were performed on tumor tissues from each group, and representative images were presented. **K** Quantitative analysis of Ki67 intensity in (**J**) and quantitative analysis of vascular density based on CD31 staining in **J** were performed. Data of **A**–**E** were represented as mean ± SEM from three independent experiments. Statistical analysis was performed using two-way ANOVA (**B**, MTT data of **D** and **E**, **F**) and Student’s *t*-test (**C**, colony assay of **D** and **E**, **H**, **K**). *P < 0.05, **P < 0.01, ***P < 0.001.

We proceeded to investigate the impact of FAM210B on LUAD cell proliferation. Given that FAM210B expression is higher in A549 cells than in H1299 cells, we knocked down FAM210B in A549 cells. Figure 2A shows the successful knockdown of FAM210B in A549 cells. The MTT assay revealed that silencing FAM210B significantly enhanced A549 cell proliferation (Fig. 2B). Similarly, the colony formation assay demonstrated a notable increase in the number of A549 cell colonies following FAM210B knockdown (Fig. 2C). Conversely, stable overexpression of FLAG-FAM210B in H1299 cells led to decreased cellular viability and colony forming capacity (Fig. 2D). Similar results were also obtained in A549 cells (Fig. 2E), with a more pronounced decrease in cell viability on day 5 compared to H1299 cells, possibly due to the stronger suppressive effect of FAM210B overexpression on A549 cell viability.

In vivo experiments showed that stable overexpression of FAM210B in both H1299 and A549 cells inhibited tumor growth in xenograft mouse models, resulting in a reduction in tumor volume (Fig. 2F–I, Fig. S2A–D). Immunohistochemistry analysis revealed a downregulation of both the proliferation marker Ki67 and the endothelial marker CD31 (indicating vascular density) from tumor tissues of A549 and H1299 when overexpressing FAM210B (Fig. 2J, K, Fig. S2E), suggesting that FAM210B suppressed xenograft tumor growth and angiogenesis.

### FAM210B suppressed the migration and invasion of LUAD cells in vitro and the tumor metastasis of LUAD tumor in vivo

**Fig. 3:**
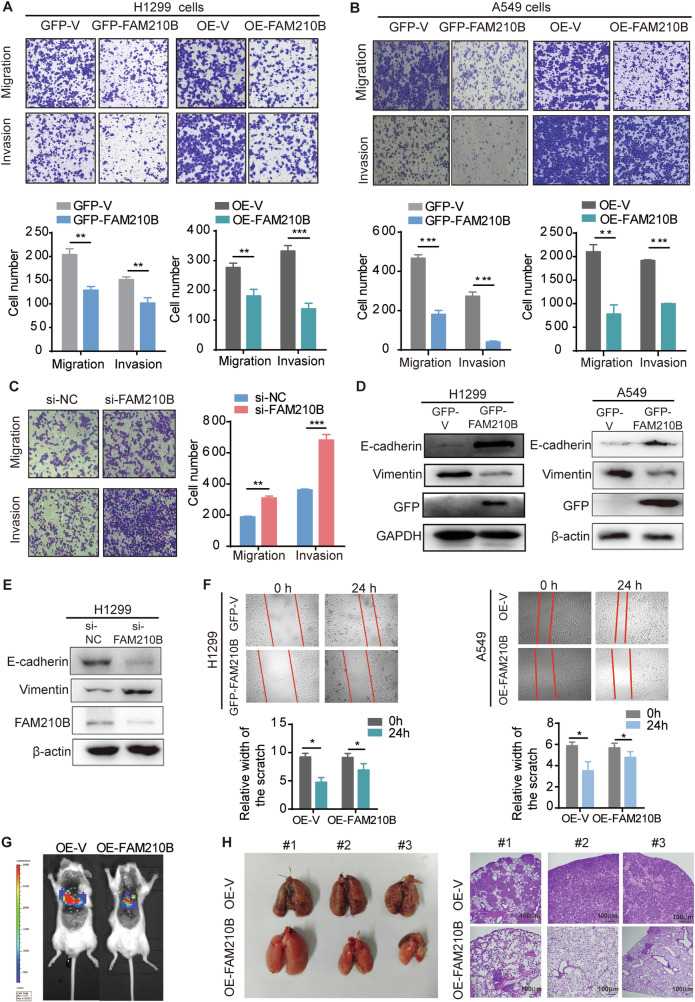
FAM210B suppressed the migration and invasion of LUAD cells in vitro and the tumor metastasis of LUAD tumor in vivo*.* **A**, **B** Transwell assays were conducted to analyze the migration and invasion abilities of H1299 cells or A549 cells transiently (left) or stably (right) overexpressing FAM210B (upper). The quantitative analysis of invasion and migration in H1299 and A549 cells was shown in the lower panels. **P < 0.01, ***P < 0.001 (Student’s *t*-test). **C** Transwell assays were performed in FAM210B knocked-down H1299 cells (left). The quantitative analysis of invasion and migration in left panel was shown in the right panel. **P < 0.01, ***P < 0.001 (Student’s *t*-test). **D**, **E** Western blot was performed on H1299 and A549 cells transiently transfected with GFP-FAM210B or FAM210B siRNA to analyze the expression of EMT markers. **F** Wound healing experiments were conducted on H1299 cells transiently overexpressing GFP-FAM210B and on A549 cells stably overexpressing FAM210B. The quantitative analysis of the scratch width was presented below the corresponding images (mean ± SEM, *n* = 3). *P < 0.05 (Student’s *t*-test). **G** Representative bioluminescent images of NCG mice acquired 40 days after tail intravenous injection of A549 cells expressing OE-V-Luc or OE-FAM210B-Luc. **H** Pictures of lungs taken from the indicated groups of nude mice (left), and histological examination of lung tissues using HE staining (right). Scale bar: 100 μm.

Next, we tested the impact of FAM210B on LUAD metastasis ability. Both transient and stable overexpression of FAM210B in H1299 and A549 cells led to a significant decrease in cell migration and invasion (Fig. 3A, B). Conversely, knockdown of FAM210B enhanced the migratory and invasive capabilities of H1299 cells (Fig. 3C). Consistently, FAM210B overexpression led to increased expression of E-cadherin (an epithelial marker) and decreased expression of Vimentin (mesenchymal markers), whereas FAM210B silencing had the opposite effect on E-cadherin and Vimentin (Fig. 3D, E). These findings were further supported by wound healing assays, demonstrating delayed wound closure in A549 and H1299 cells overexpressing FAM210B (Fig. 3F).

Metastasis suppression by FAM210B was then validated in the tail vein injection model, as evidenced by the reduced fluorescence intensity observed in OE-FAM210B group compared to control group (Fig. 3G). The A549-injected mice had shrunk lungs covered by pulmonary metastatic nodules, while those of mice injected with A549 cells overexpressing FAM210B displayed normal form and much smoother surfaces (Fig. 3H, left panel). Furthermore, HE staining demonstrated decreased tumor metastasis area in lung tissues for the FAM210B overexpressing group compared to the control group (Fig. 3H, right panel). These results indicated that FAM210B inhibited metastasis ability of LUAD.

### FAM210B activated the STAT1/IRF9/IFIT3 signal pathway through IFN-α/β upregulation

**Fig. 4:**
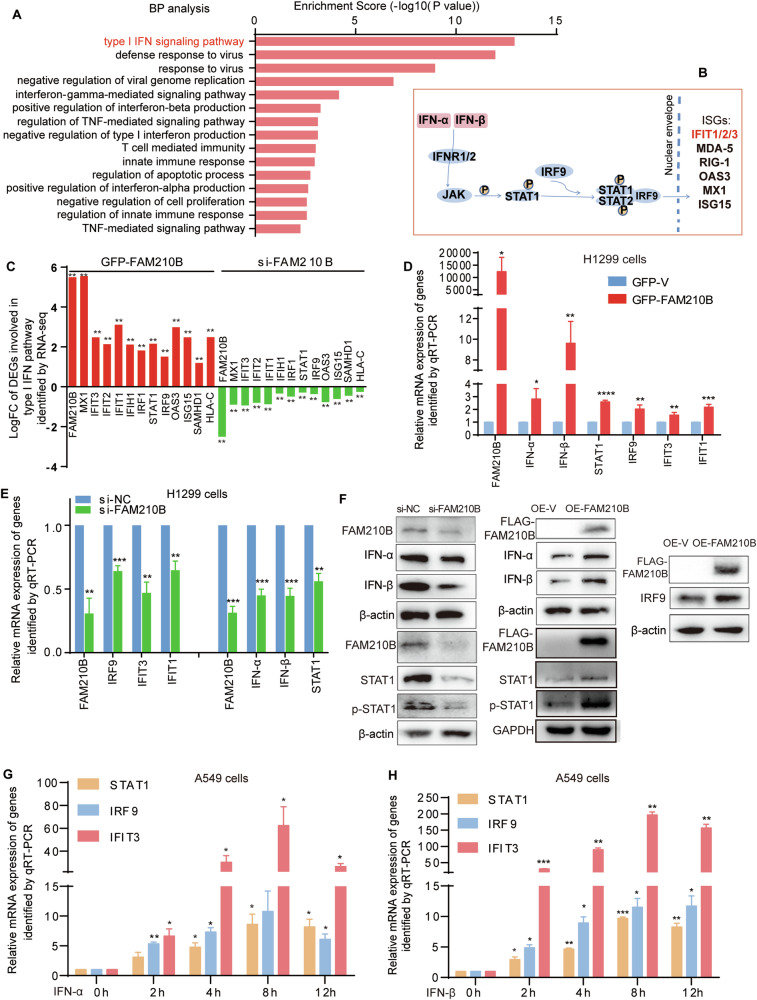
FAM210B activated the IFN-α/β mediated STAT1/IRF9/IFIT3 signal pathway. **A** GO Biological Process (BP) analysis of 113 DEGs regulated by FAM210B, with the top 15 BPs listed. **B** A schematic representation of the type I IFN signaling pathway. The circled P represents phosphate. **C** Log (fold change) of mRNA expression level of DEGs involved in the type I IFN signaling pathway, as assayed by RNA-seq with FAM210B overexpressing or knockdown H1299 cells. All the selected genes have a *P* value of less than 0.01 (**P < 0.01). qRT-PCR assays were used to test the expression levels of several representative members of the type I IFN signaling pathway, along with IFN-α and IFN-β, in H1299 cells overexpressing GFP-FAM210B (**D**) or following FAM210B knockdown (**E**) (mean ± SEM, *n* = 3). **F** Western blotting was conducted in H1299 cells with FAM210B knockdown or overexpression (OE-FAM210B) to evaluate changes in representative members of the type I IFN signaling pathway. **G**–**H** qRT-PCR assays were employed to assess the expression levels of STAT1, IRF9, and IFIT3 in LUAD cells under treatment with IFN-α or IFN-β at different time points (*n* = 2), respectively. Statistical analysis was performed using Student’s *t*-test. *P < 0.05, **P < 0.01, ***P < 0.001, ****P < 0.0001.

To explore the mechanism underlying FAM210B’s functions, we performed an RNA-seq assay with H1299 cells in which FAM210B was either overexpressed or knocked down. A total of 113 differentially expressed genes (DEGs) were identified based on their opposite differential expression in FAM210B-overexpressed and -knocked-down cells (Table S3, *P* < 0.01). Strikingly, most of the top 15 GO-BP terms associated with these genes concerned IFN-mediated signal pathway, IFN production, and innate immune response (Fig. 4A).

IFN-α/β is well known to activate JAK1/STAT1/IRF9 signal pathway to promote the transcription of ISG, such as IFIT1-3 [4, 5, 26, 27] (Fig. 4B). Our data of RNA-seq assay showed that within this pathway, the mRNA expression levels of 12 key factors were altered by FAM210B overexpression and knockdown (Fig. 4C). Four representative genes of this pathway, STAT1, IRF9, IFIT1, and IFIT3, along with two key upstream cytokines, IFN-α and IFN-β, were identified to be positively regulated by FAM210B, as demonstrated by qRT-PCR assays in FAM210B-overexpressed and FAM210B-knockdown cells (Fig. 4D, E). Western blotting assays revealed a similar trend of regulation for IFN-α, IFN-β, STAT1, and IRF9 by FAM210B, as well as the positive regulation of p-STAT1 by FAM210B (Fig. 4F). Moreover, treating LUAD cells with 60 ng/mL IFN-α or IFN-β stimulated the mRNA expression of STAT1, IRF9, and IFIT3 in a time-dependent manner (Fig. 4G, H). These results suggested that FAM210B promotes the expression of IFN-α/β and activates the IFN-α/β mediated STAT1/IRF9/IFIT3 signaling pathway.

### FAM210B inhibited the proliferation and migration of LUAD cells via STAT1/IRF9/IFIT3 axis

**Fig. 5:**
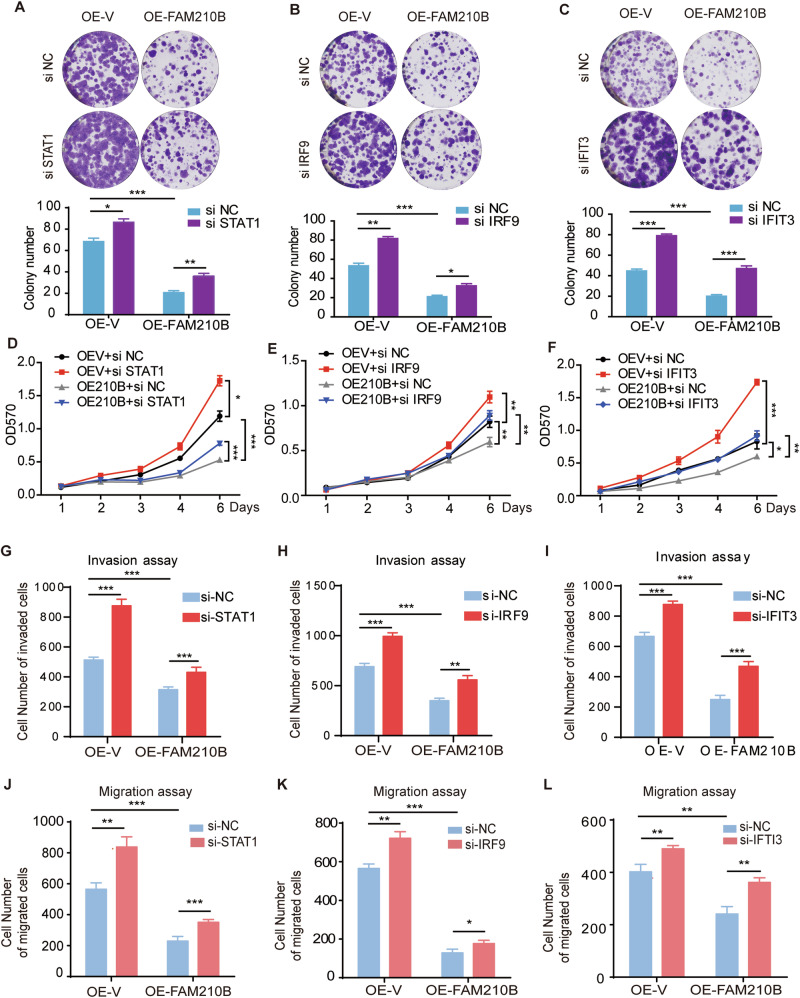
STAT1/IRF9/IFIT3 axis was involved in the inhibitory function of FAM210B in the proliferation and migration of LUAD cells. Colony formation assay of OE-V and OE-FAM210B H1299 cells transfected with si-STAT1 (**A**), si-IRF9 (**B**), or si-IFIT3 (**C**) (top panels). Quantitative analysis of these colony formation of three biological replicates was shown in bottom panels. MTT assays of OE-V and OE-FAM210B H1299 cells transfected with si-STAT1 (**D**), si-IRF9 (**E**), or si-IFIT3 (**F**). Quantitative analysis of transwell assays for invasion and migration in OE-V and OE-FAM210B H1299 cells transfected with si-STAT1(**G**, **J**), si-IRF9 (**H**, **K**), or si-IFIT3 (**I**, **L**) as indicated. All data were represented as mean ± SEM from three independent experiments. Statistical analysis was performed using two-way ANOVA (**D**, **E**, **F**) and Student’s *t*-test (**A**–**C**, **G**–**L**). *P < 0.05, **P < 0.01, ***P < 0.001.

We further explored whether the IFN-α/β-activated STAT1/IRF9/IFIT3 axis participated in FAM210B’s biological functions. Knocking down STAT1, IRF9, or IFIT3 promoted colony formation and more importantly countered the effect of FAM210B overexpression (Fig. 5A–C). Similar results were also obtained in MTT assays (Fig. 5D–F). Finally, transwell assays also demonstrated that knockdown of STAT1, IRF9, or IFIT3 partially reversed the downregulation of invasion and migration induced by FAM210B overexpression (Fig. 5G–L, Fig. S3A–I). Taken together, these results suggested that the function of FAM210B was mediated by STAT1/IRF9/IFIT3 axis in LUAD cells.

### FAM210B induced the expression of IFN-α/β through interacting with TOM70 and activating IRF3

**Fig. 6:**
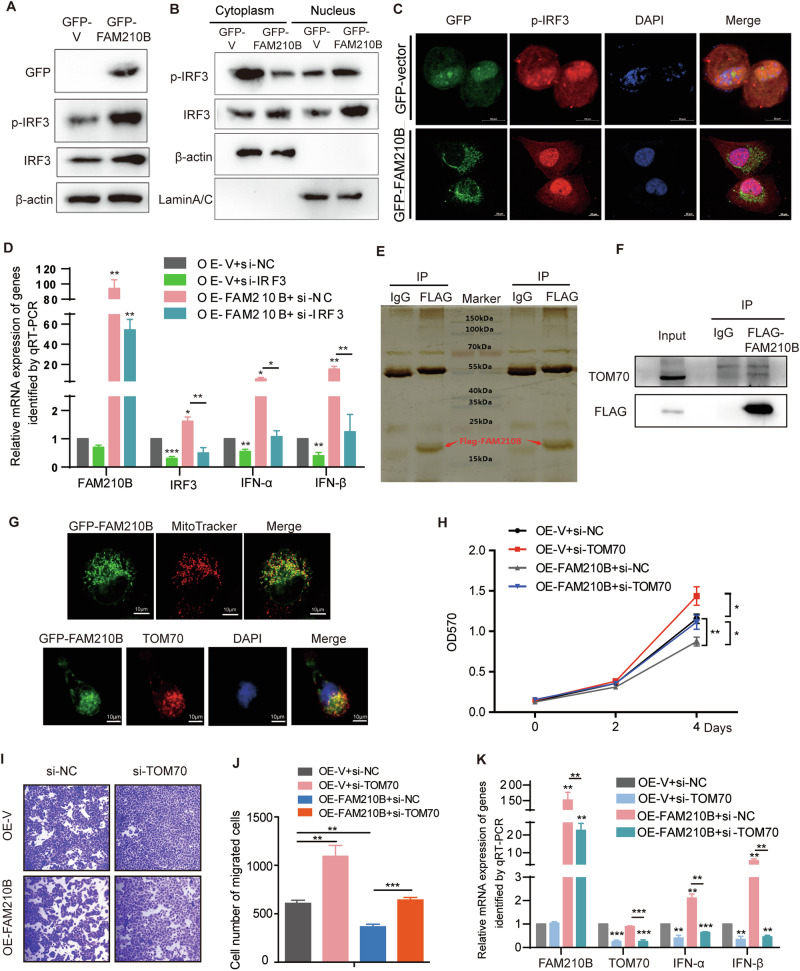
FAM210B promoted the expression of IFN-α/β via activating IRF3 and associating with TOM70. **A** Western blot assay of the expression of p-IRF3 and IRF3 in H1299 cells transfected with GFP-V or GFP-FAM210B. **B** Nuclear-cytoplasmic separation assays were used to analyze the expression changes of p-IRF3 in both nuclear and cytoplasmic fractions in H1299 cells transfected with GFP-V or GFP-FAM210B. **C** IF assay of localization of p-IRF3 in H1299 cells transfected with GFP-V or GFP-FAM210B. Scale bar: 10 μm. **D** qRT-PCR assays of the indicated genes in OE-V and OE-FAM210B H1299 cells combined with si-IRF3 as indicated. **E** Silver staining of the immunocomplex immunoprecipitated by FLAG antibody and non-immune IgG antibody. **F** The interaction of FLAG-FAM210B with TOM70 confirmed by co-IP assay in H1299 OE-FAM210B cells. **G** Imaging assay was used to assess the colocalization of GFP-FAM210B with mitochondria (MitoTracker Red) in live H1299 cells (upper), and IF assay was performed to detect the colocalization of GFP-FAM210B with TOM70 in H1299 cells transfected with GFP-FAM210B (below). Scale bar: 10 μm. **H** MTT assay of the cellular viability of OE-V and OE-FAM210B H1299 cells transfected with si-TOM70. **I** Transwell assays of the migration of OE-V and OE-FAM210B H1299 cells transfected with si-TOM70. The corresponding quantitative analysis of this transwell assay is shown in (**J**). **K** qRT-PCR assay of the expression of IFN-α/β in OE-V and OE-FAM210B H1299 cells transfected with si-TOM70. All quantitative analyses are presented as mean ± SEM from three independent experiments. Statistical analysis was conducted using Student’s *t*-test (**D**, **J**, **K**) and two-way ANOVA (H). *P < 0.05, **P < 0.01, ***P < 0.001.

It has been reported that transcription factor IRF3 plays a crucial role in the transcription of IFN-α/β, so we examined if FAM210B regulated IFN-α/β expression through IRF3. Our results revealed that overexpressing FAM210B increased the levels of phosphorylated IRF3 and total IRF3, and facilitated the nuclear localization of phosphorylated IRF3 as examined by nuclear-cytoplasmic separation and IF assays (Fig. 6A–C). Consistently, knocking down IRF3 lowered the transcriptional expression of IFN-α/β and suppressed FAM210B-induced upregulation of IFN-α/β mRNA level (Fig. 6D). These findings indicated that FAM210B indeed stimulated the transcriptional expression of IFN-α/β through regulation of IRF3 expression, phosphorylation and nuclear import.

It has been reported that FAM210B was localized in the mitochondrial membrane [15], and mitochondrial proteins were crucial for producing IFN-α/β [28]. Using immunoprecipitation of the overexpressed FLAG-FAM210B in H1299 cells (Fig. 6E) followed by Mass Spectrometry analysis, we have identified TOM70 (Translocase of outer mitochondrial membrane 70, also named as TOMM70), a mitochondrial outer membrane protein as a potential partner of FAM210B (Table S4). This interaction was further confirmed by conventional co-IP assay (Fig. 6F). IF assay also demonstrated the co-localization of FAM210B with mitochondria, as well as its co-localization with TOM70 in the mitochondria of LUAD cells (Fig. 6G). Interestingly, TOM70 silencing exhibited promoting effect on proliferation and migration of LUAD cells and reversed the inhibitory effect of FAM210B overexpression on these phonotypes (Fig. 6H–J). Consistently, TOM70 knockdown inhibited the increased expression of IFN-α/β in OE-FAM210B cells (Fig. 6K). Our data suggested that the interaction between FAM210B and TOM70 promoted the expression of IFN-α/β and thereby inhibited the proliferation and migration of LUAD cells. Interestingly, we found that TOM70 knockdown led to a reduction of FAM210B expression in cells overexpressing OE-FAM210B, whereas the overexpression of FAM210B did not affect TOM70 levels (Fig. 6K). Further, we evaluated the effects of STAT1, IRF9, and IRF3, three downstream effectors of IFN-α/β signaling, on the expression of TOM70 and FAM210B. qRT-PCR assays showed that knockdown of STAT1, IRF9, or IRF3 caused a reduction of the expression of FAM210B, whereas knockdown of STAT1, IRF9, or IRF3 caused little or no effects on TOM70 expression (Fig. S4A–C).

## Discussion

LUAD is the most prevalent histological subtype of lung cancer, characterized by high morbidity and fatality rates and a poor prognosis [1, 2]. Discovering new molecular mechanisms that contribute to LUAD tumorigenesis would provide valuable insight into identifying potential drug targets for LUAD therapy. In this study, we demonstrated both in vitro and in vivo that FAM210B significantly inhibited the growth and metastasis of LUAD cells, suggesting that FAM210B might be a promising suppressor of LUAD progression.

Through analysis of transcriptional profiles, type I IFN (IFN-α/β) signal pathway was identified as the top biological process regulated by FAM210B. Our data demonstrated that FAM210B stimulated the expression of IFN-α/β in LUAD cells and in subcutaneous tumor tissues from mice receiving OE-FAM210B cells (Fig. S5), suggesting that elevated expression of IFN-α/β might be responsible for the repressive function of FAM210B in LUAD cells. IFN-α/β has been used as a form of cytokine therapy for melanoma treatment [26, 29, 30]. Previous studies mainly focused on its anti-tumor activities through facilitating the anti-tumor immune responses by increasing immune cell infiltration of CD4^+^ and CD8^+^ T-cells [31, 32]. As comparison, the autocrine signaling of IFN-α/β was less explored [6–11]. Our research revealed a FAM210B-promoted IFN-α/β autocrine signaling pathway in LUAD cells, leading to the suppression of LUAD development.

The primary function of IFN-α/β is mediated through the activation of the JAK-STAT signal pathway [26, 27]. The role of the activated STAT1 as either a tumor suppressor or a tumor promoter has been reported [33–36]. Similarly, IRF9, whose overexpression has been reported to cause resistance of tumor cells to drugs [37, 38] and radiation [39] and exert oncogenic effects [40, 41], has also been shown to be a key regulator for mediating the antiproliferative effect of IFN-α in tumor cells elsewhere [27, 42]. In this study, we showed that the inhibitory effects of STAT1/IRF9 axis mediated the suppressive function of FAM210B on the growth, mobility, and invasion of LUAD cells, while also being involved in regulating FAM210B mRNA expression. Additionally, previous studies have shown that targeting the delivery of IFN-α via Tie2-expressing monocytes (TEMs) inhibits tumor angiogenesis [43]. Similarly, endogenous IFN-β has been reported to suppress tumor angiogenesis by downregulating pro-angiogenic factors such as VEGF and MMP9, as well as homing factors in neutrophils [44]. Moreover, STAT1 has also been shown to negatively regulate pro-angiogenic molecules like bFGF, significantly inhibiting tumorigenicity, angiogenesis, and metastasis, thereby serving as a negative regulator of tumor growth and metastasis [33]. In our findings, FAM210B overexpression promoted the expression of IFN-α/β and STAT1, inhibited EMT, a key process in tumor cell metastasis, and reduced the expression of the endothelial marker CD31 in mouse tumor tissues. Taken together, all these data might hint on the possible inhibitory role of FAM210B on tumor angiogenesis and metastasis in LUAD through IFN-α/β/ STAT1 signal axis.

**Fig. 7:**
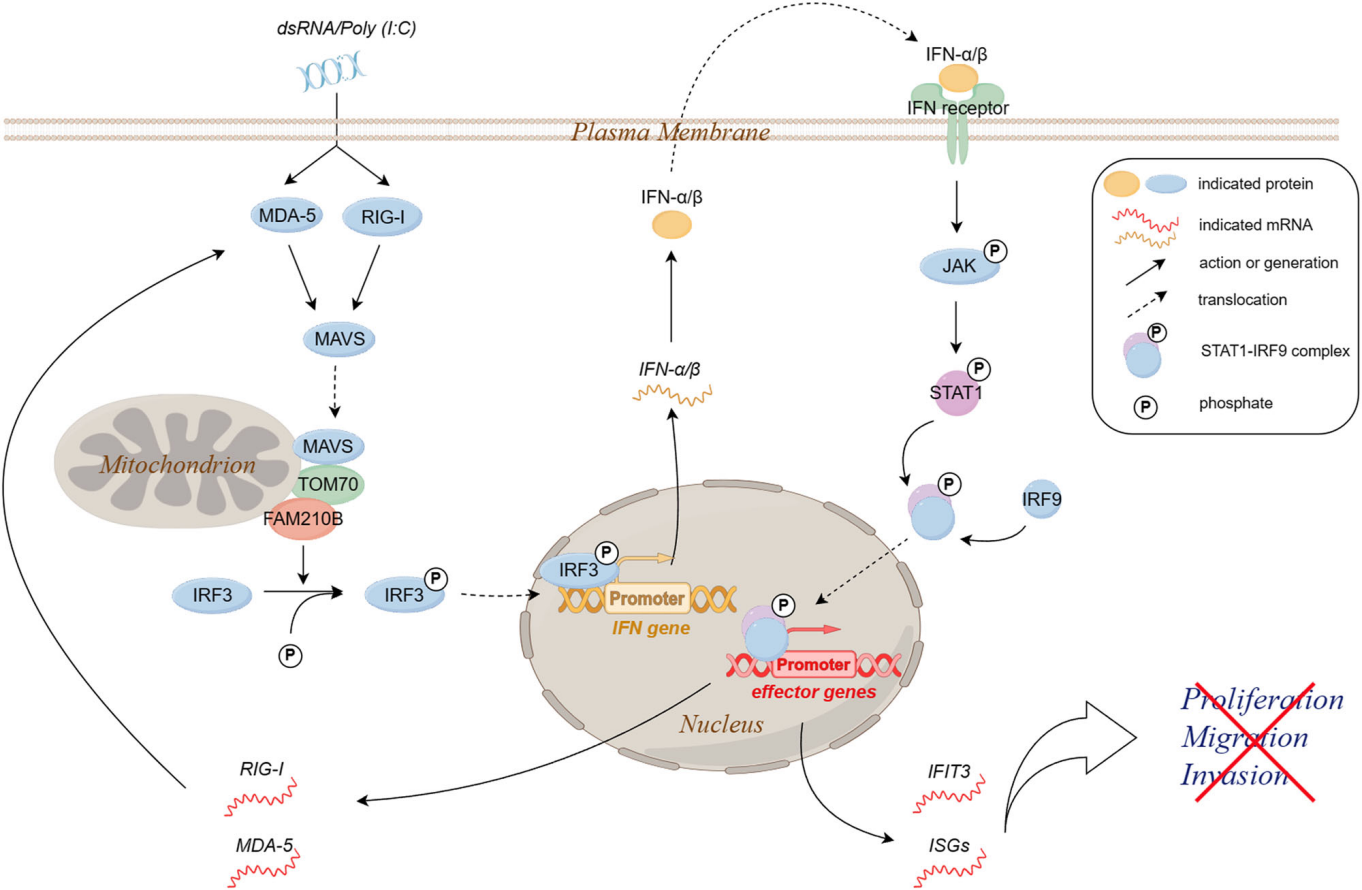
FAM210B inhibits the proliferation and metastasis of lung adenocarcinoma via interacting with TOM70 and promoting IFN-α/β production. When FAM210B is overexpressed in LUAD cells, it interacts with TOM70, leading to the phosphorylation and nuclear translocation of IRF3. This activation of IRF3 enhances the expression and secretion of IFN-α/β, which then binds to its receptor and activates the STAT1/IRF9 pathway. As a result, the expression of ISGs like IFIT3, MDA-5, and RIG-I is induced, which in turn suppresses LUAD cell proliferation, migration, and invasion. Elevated levels of MDA-5 and RIG-I further stimulate the innate immune response, with FAM210B playing a role in amplifying IFN-α/β expression.

In this study, we identified TOM70, a 70-kDa mitochondrial membrane-anchored adapter, as an interacting partner of FAM210B. TOM70 plays a crucial role in innate immunity by acting as an adaptor to link mitochondrial antiviral signaling protein (MAVS) to TANK-binding kinase 1 (TBK1)/IRF3 activation [28], which is essential for the induction of type I IFN [45–47]. We observed that TOM70 and FAM210B promoted IFN-α/β production and inhibited LUAD cell proliferation and migration. These findings depicted a previously unknown signaling pathway in which TOM70 interacts with FAM210B to participate in the upregulation of IFN-α/β expression, and characterized FAM210B as a novel regulator of innate immune response (Fig. 7). Considering the absence of MAVS in the FAM210B-interactor list in our interactome study, a direct and stable interaction between FAM210B and MAVS might be excluded. However, given our findings that FAM210B not only stimulated the expression of two upstream regulators of MAVS, namely MDA-5 and RIG-I (Fig. S6A, B) but also interacted with TOM70, a binding protein of MAVS, cooperative functional links could exist between FAM210B and MAVS in the signaling transduction, which, with the complexity provided by the participation of TOM70 and other factors, might need further investigation in the future to be elucidated.

Our data also indicated that both FAM210B overexpression and the treatment of LUAD cells with purified IFN-α protein enhanced the expression of MDA-5 and RIG-I which are upstream regulators in the innate immune pathway (Fig. S6A–D). Moreover, knockdown of MDA-5 in FAM210B-overexpressing cells abolished the upregulation of IFN-α/β caused by FAM210B overexpression (Fig. S6E). Based on these findings, we hypothesize that a positive feedback loop may take place: FAM210B promotes the expression of IFN-α/β, which in turn induces the expression of MDA-5 and RIG-I. The increased expression of MDA-5 and RIG-I then stimulates the innate immune pathway, in which FAM210B participates to further promote the expression of IFN-α/β (Fig. 7). Such mechanism might have important implication for future therapeutic applications based on the anti-tumor properties of FAM210B.

## Conclusion

In summary, we demonstrated the relevance of the novel protein FAM210B to LUAD development, supported by the positive correlation between FAM210B expression and the overall survival of LUAD patients. FAM210B was found to inhibit LUAD growth and metastasis both in vitro and in vivo. Mechanistically, FAM210B interacted with TOM70, leading to the upregulation of IFN-α/β expression and activation of the innate immune pathway in LUAD cells. The IFN-α/β-activated STAT1/IRF9/IFIT3 axis played a crucial role in suppressing the proliferation and metastasis of LUAD cells. These findings offer new insights into LUAD pathogenesis and suggest that targeting the FAM210B/IFN-α/β/STAT1/IRF9/IFIT3 axis may hold promise as a therapeutic strategy for LUAD.

## Supplementary information

Supplementary File 1

Supplementary Table S1

Supplementary Table S2

Supplementary Table S3

Supplementary Table S4

Original data

## Data Availability

All data generated and analyzed during this study are included in this article and its Supplementary files. The data and materials used in this study are available upon reasonable request to the corresponding author.
