# Supplementary material for: FAM210B activates STAT1/IRF9/IFIT3 axis by upregulating IFN-α/β expression to impede the progression of lung adenocarcinoma
Source: Cell Death Dis. 2025 Feb 3;16(1):63. doi: 10.1038/s41419-025-07375-9 (PMC11791038; doi:10.1038/s41419-025-07375-9)
Supplement: Supplementary file 1 [file 41419_2025_7375_MOESM1_ESM.docx]

Supplementary file 1


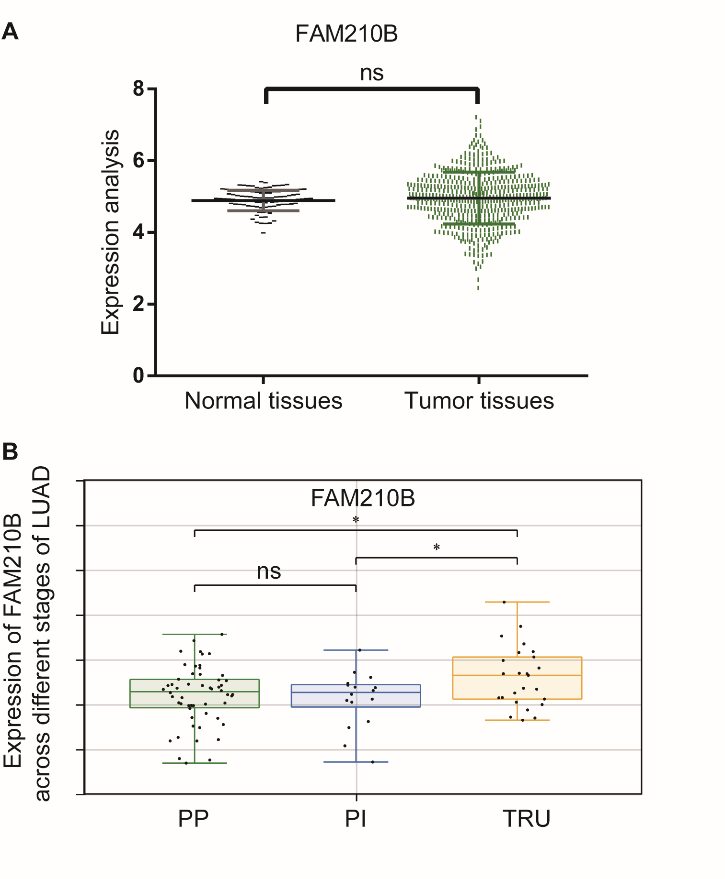


**Fig. S1** The expression of FAM210B in LUAD patients.

(A) Gene expression of FAM210B in normal and tumor tissues of LUAD patients. The TCGA data for LUAD samples was downloaded from Sangerbox (http://vip.sangerbox.com/login.html), which includes 513 LUAD tumor samples and 109 normal samples. An unpaired t-test was used for statistical analysis. (B) FAM210B expression across different stages of LUAD. TRU: terminal respiratory unit, PI: proximal inflammatory, PP: proximal proliferative. ns: not significant, **P<*0.05 (Student's *t*-test).


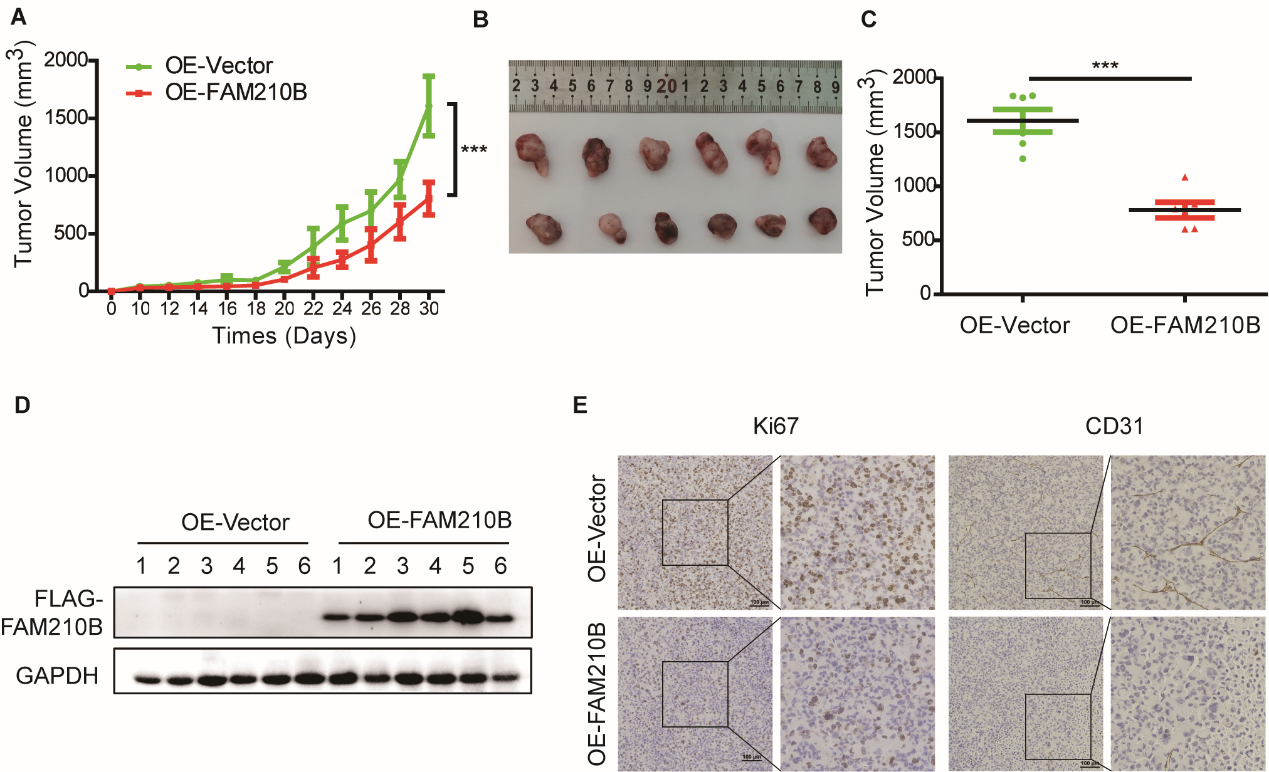


**Fig. S2** FAM210B suppressed tumor growth from H1299 cells *in vivo.* (A-C) The experiments involved the injection of either OE-FAM210B H1299 cells or OE-Vector H1299 cells. The results obtained from the respective nude mouse groups include: measurement of tumor volume at different time points (A, at 30 days after subcutaneous injection of H1299 OE-V and OE-FAM210B cells, the measured tumor volume approached the upper limit allowed by animal ethics guidelines, so we terminated the experiment), images of tumors at the endpoints of time (B), and statistical analysis of tumor volumes (C). Statistical analysis was performed using two-way ANOVA (A) and Student's *t*-test (C). ****P<*0.001. (D) Western blot assays were performed on the tumor tissues from the indicated nude mouse groups to confirm the overexpression of FAM210B. (E) IHC analyses of Ki67 and CD31 were performed on tumor tissues from each group, with representative images provided.


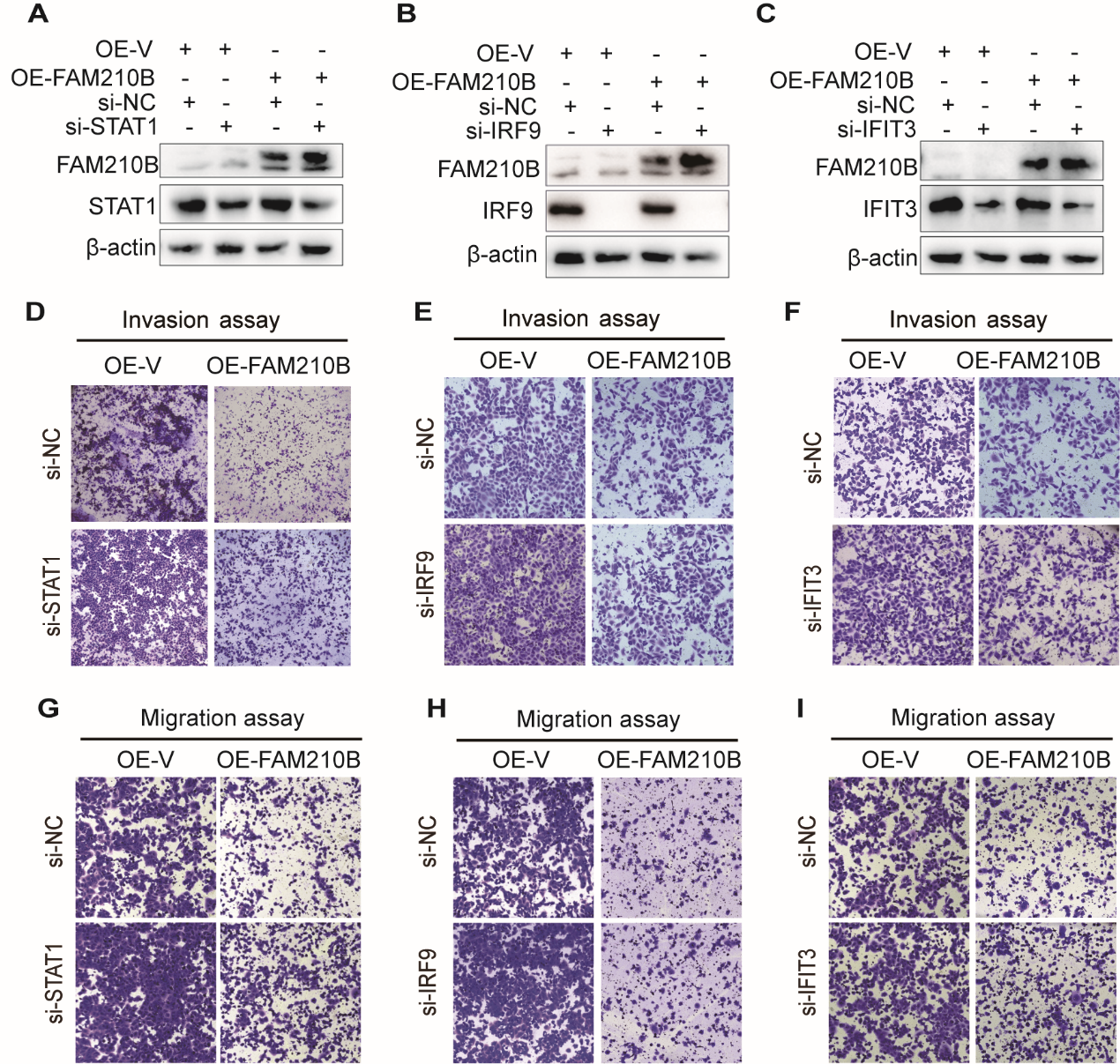


**Fig. S3** The STAT1/IRF9/IFIT3 axis was involved in the inhibitory role of FAM210B in the invasion and migration of LUAD cells. Western blot assays were performed to detect the expression levels of STAT1, IRF9, and IFIT3 in OE-FAM210B cells following knockdown of STAT1 (A), IRF9 (B), or IFIT3 (C), as indicated. Transwell assays were conducted to assess invasion and migration in OE-V and OE-FAM210B H1299 cells transfected with si-STAT1 (D, G), si-IRF9 (E, H), or si-IFIT3 (F, I), as indicated.


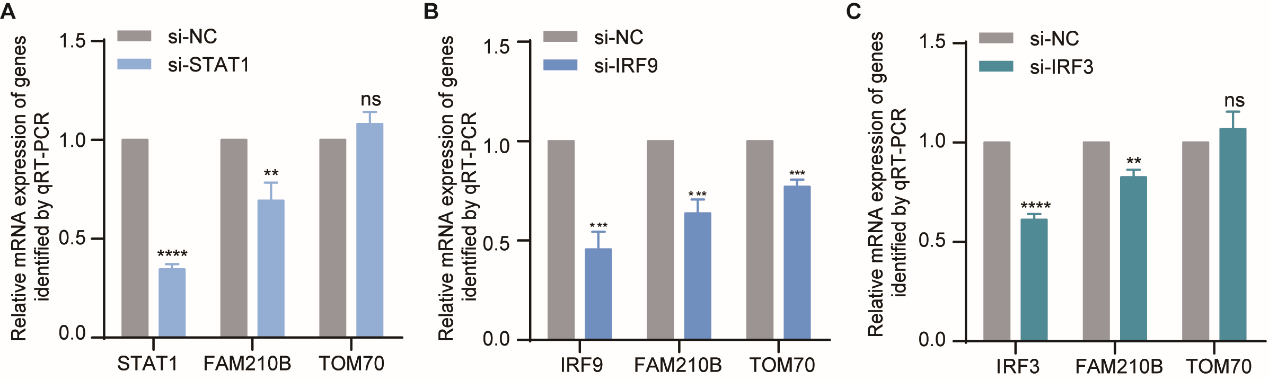


**Fig. S4** The effects of STAT1, IRF9, and IRF3 on the expressions of FAM210B and TOM70. qRT-PCR analysis of the mRNA expression levels of FAM210B, TOM70, and STAT1 (A), FAM210B, TOM70, and IRF9 (B), and FAM210B, TOM70, and IRF3 (C) in H1299 cells transfected with si-STAT1 (A), si-IRF9 (B), or si-IRF3 (C). Data were represented as mean ± SEM from three independent experiments. ns: not significant, ***P*<0.01, ****P*<0.001, *****P*<0.0001 (Student's *t*-test).


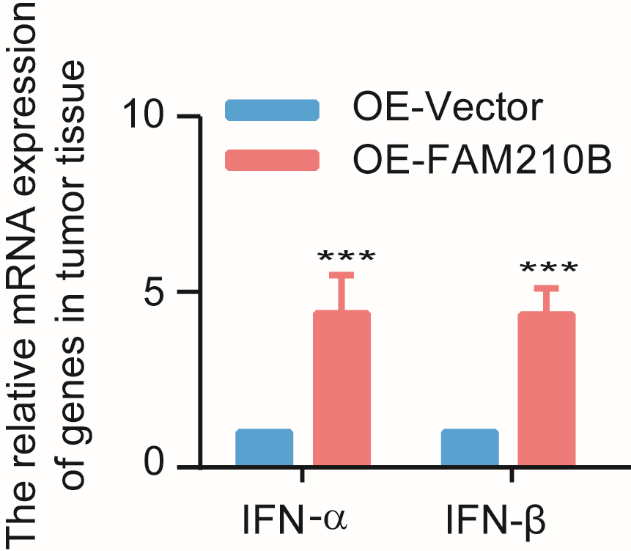


**Fig. S****5** qRT-PCR assay of the expression of IFN-α/β in tumor tissues from nude mice injected with either OE-V or OE-FAM210B A549 cells (n=6). ****p*<0.001 (Student's *t*-test).


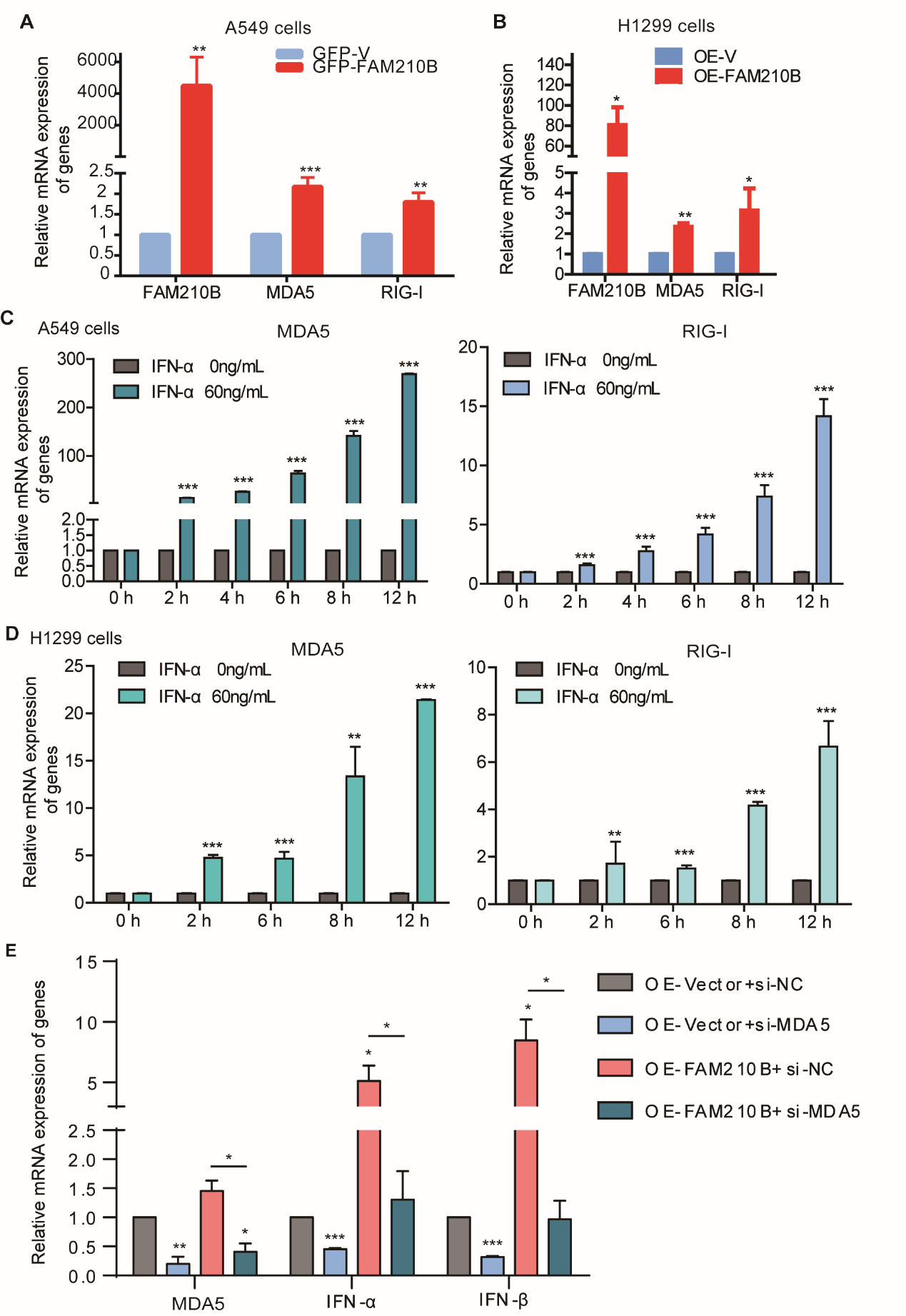


**Fig. S6** The effects of FAM210B on the expressions of MDA-5 and RIG-I. (A-B) qRT-PCR assay of the expressions of MDA-5 and RIG-I in LUAD cells transfected with GFP-V or GFP-FAM210B or stably expressing vector (OE-V) or FAM210B (OE-FAM210B). (C-D) qRT-PCR assay of the expressions of MDA-5 and RIG-I in LUAD cells after treatment with IFN-α. (E) qRT-PCR assay of the expressions of MDA-5 and IFN-α/β in OE-V and OE-FAM210B H1299 cells transfected with si-MDA-5. Data were represented as mean ± SEM from three independent experiments. **P*<0.05, ***P*<0.01, ****P*<0.001 (Student's *t*-test).
