# Supplementary material for: FAM210B activates STAT1/IRF9/IFIT3 axis by upregulating IFN-α/β expression to impede the progression of lung adenocarcinoma
Source: Cell Death Dis. 2025 Feb 3;16(1):63. doi: 10.1038/s41419-025-07375-9 (PMC11791038; doi:10.1038/s41419-025-07375-9)
Supplement: Supplementary file 4 — Supplementary Table S3 [file 41419_2025_7375_MOESM4_ESM.docx]

**Table S3** 113 differential genes with a *P*-value of less than 0.01 identified by RNA-seq assay on both FAM210B overexpressing (OE-FAM210B) and knocking down (si-FAM210B) H1299 cells.

| **Gene Names** | **Official Full Name** | **LogFC (OE-FAM210B vs OE-V)** | **LogFC (si-FAM210B vs si-NC)** | **MW [kDa]** | **P Value** |
| --- | --- | --- | --- | --- | --- |
| MX1 | MX dynamin like GTPase 1 | 5.554619204 | -0.892865087 | 72.82 | 5.16E-21 |
| FAM210B | family with sequence similarity 210 member B | 5.49987493 | -2.501650751 | 21.12 | 3.23E-121 |
| SAMD9L | sterile alpha motif domain containing 9 like | 4.174355671 | -1.154030204 | 174.24 | 2.37E-22 |
| GBP1 | guanylate binding protein 1 | 4.019669662 | -1.052460203 | 65.12 | 1.12E-19 |
| PARP10 | poly(ADP-ribose) polymerase family member 10 | 3.856543823 | -0.810671567 | 114.07 | 1.93E-16 |
| IFI6 | interferon alpha inducible protein 6 | 3.377989722 | -0.429040864 | 15.18 | 4.23E-13 |
| USP18 | ubiquitin specific peptidase 18 | 3.342537997 | -0.544171702 | 40.92 | 4.90E-24 |
| LGALS3BP | galectin 3 binding protein | 3.239619622 | -0.719209132 | 64.35 | 7.97E-20 |
| SERPING1 | serpin family G member 1 | 3.23528006 | -0.481191602 | 55 | 2.12E-15 |
| IFIT1 | interferon induced protein with tetratricopeptide repeats 1 | 3.115241218 | -0.868982408 | 52.58 | 3.01E-21 |
| OAS3 | 2'-5'-oligoadenylate synthetase 3 | 2.985246242 | -0.770322905 | 119.57 | 2.53E-19 |
| PARP9 | poly(ADP-ribose) polymerase family member 9 | 2.685341137 | -0.455558636 | 93.94 | 5.17E-21 |
| SAMD9 | sterile alpha motif domain containing 9 | 2.634475664 | -0.812313359 | 174.79 | 3.00E-21 |
| HELZ2 | helicase with zinc finger 2 | 2.62445139 | -0.60167826 | 291.39 | 1.33E-15 |
| HLA-C | major histocompatibility complex, class I, C | 2.483627224 | -0.2580241 | 40.26 | 5.49E-20 |
| IFIT3 | interferon induced protein with tetratricopeptide repeats 3 | 2.4790496 | -0.937202475 | 53.9 | 8.19E-18 |
| ISG15 | ISG15 ubiquitin like modifier | 2.475324677 | -0.61391249 | 18.15 | 9.65E-22 |
| STAT1 | signal transducer and activator of transcription 1 | 2.159060622 | -0.287753107 | 82.5 | 6.05E-18 |
| IFIT2 | interferon induced protein with tetratricopeptide repeats 2 | 2.133976509 | -0.802120768 | 51.92 | 5.68E-16 |
| IFIH1 | interferon induced with helicase C domain 1 | 2.11811945 | -0.335742304 | 112.75 | 7.60E-19 |
| PARP14 | poly(ADP-ribose) polymerase family member 14 | 2.10848988 | -0.396539847 | 198.11 | 5.96E-19 |
| DDX58 | RNA sensor RIG-I | 2.107227938 | -0.459189703 | 101.53 | 4.66E-15 |
| TDRD7 | tudor domain containing 7 | 1.930799943 | -0.395633345 | 120.78 | 1.54E-18 |
| IRF1 | interferon regulatory factor 1 | 1.823724433 | -0.485564073 | 35.75 | 3.26E-14 |
| BTN3A3 | butyrophilin subfamily 3 member A3 | 1.75242509 | -0.623453301 | 64.24 | 2.14E-09 |
| HIST2H2AA4 | H2A clustered histone 19 | 1.709634095 | 0.507514752 | 14.3 | 1.50E-18 |
| IL22RA1 | interleukin 22 receptor subunit alpha 1 | 1.694764467 | -1.25474793 | 63.14 | 3.67E-09 |
| HERC6 | HECT and RLD domain containing E3 ubiquitin protein ligase family member 6 | 1.558544047 | -0.37175214 | 112.42 | 3.98E-15 |
| TENT5A | terminal nucleotidyltransferase 5A | 1.527878645 | -0.546674922 | 48.62 | 7.36E-15 |
| IRF9 | interferon regulatory factor 9 | 1.508760177 | -0.365021426 | 43.23 | 3.33E-11 |
| EIF2AK2 | eukaryotic translation initiation factor 2 alpha kinase 2 | 1.461276197 | -0.393200048 | 60.61 | 2.89E-15 |
| TNFRSF1B | TNF receptor superfamily member 1B | 1.456683419 | -0.989430033 | 50.71 | 1.50E-10 |
| DDX60 | DExD/H-box helicase 60 | 1.43967434 | -0.399677337 | 188.32 | 1.22E-12 |
| MDGA1 | MAM domain containing glycosylphosphatidylinositol anchor 1 | 1.427352201 | -0.961178414 | 105.05 | 0.000000453 |
| BTN3A1 | butyrophilin subfamily 3 member A1 | 1.392546541 | -0.601135498 | 56.43 | 3.42E-10 |
| MLKL | mixed lineage kinase domain like pseudokinase | 1.383353614 | -0.5754161 | 51.81 | 1.43E-10 |
| BTN3A2 | butyrophilin subfamily 3 member A2 | 1.249730422 | -0.6094828 | 36.74 | 3.04E-11 |
| SAMHD1 | SAM and HD domain containing deoxynucleoside triphosphate triphosphohydrolase 1 | 1.199239334 | -0.447183177 | 68.86 | 1.04E-17 |
| ZC3HAV1 | zinc finger CCCH-type containing, antiviral 1 | 1.146462807 | -0.391625555 | 99.22 | 2.98E-15 |
| SDSL | serine dehydratase like | 1.140233419 | 0.566812845 | 36.19 | 4.63E-08 |
| HIST1H2AC | H2A clustered histone 6 | 1.130471781 | 0.473999285 | 14.3 | 6.45E-14 |
| B3GNT3 | UDP-GlcNAc:betaGal beta-1,3-N-acetylglucosaminyltransferase 3 | 1.106055196 | -0.762762177 | 40.92 | 0.001973336 |
| DSP | desmoplakin | 1.054352216 | -0.375679545 | 315.81 | 2.84E-13 |
| RNF213 | ring finger protein 213 | 1.031996498 | -0.381751131 | 572.77 | 0.000000178 |
| IL7R | interleukin 7 receptor | 0.996755337 | -1.035534124 | 50.49 | 0.006223373 |
| GRIN2B | glutamate ionotropic receptor NMDA type subunit 2B | 0.969542603 | -0.972268148 | 163.24 | 0.0000556 |
| BIRC3 | baculoviral IAP repeat containing 3 | 0.911484749 | -0.924749359 | 66.44 | 0.00000378 |
| SSTR2 | somatostatin receptor 2 | 0.893045594 | -1.034278483 | 40.59 | 0.007355792 |
| FAM43A | family with sequence similarity 43 member A | 0.866613711 | -0.832755828 | 46.53 | 0.0000975 |
| NID2 | nidogen 2 | 0.835220067 | -0.426188397 | 151.25 | 0.000023 |
| ID3 | inhibitor of DNA binding 3 | 0.802551135 | -0.516702164 | 13.09 | 0.000581303 |
| PLK2 | polo like kinase 2 | 0.79529419 | -0.474059358 | 75.35 | 0.00000181 |
| TNFRSF9 | TNF receptor superfamily member 9 | 0.739922068 | -1.309009163 | 28.05 | 0.000975847 |
| PRRG4 | proline rich and Gla domain 4 | 0.737527966 | -1.005875663 | 24.86 | 0.001457323 |
| ZNF204P | zinc finger protein 204, pseudogene | 0.729587836 | -0.586268903 | NA | 0.000151008 |
| OAF | out at first homolog | 0.726746151 | -0.523326029 | 30.03 | 0.0000736 |
| SPSB1 | splA/ryanodine receptor domain and SOCS box containing 1 | 0.716160377 | -0.513894897 | 30.03 | 0.00000572 |
| MASTL | microtubule associated serine/threonine kinase like | 0.668058566 | -0.305952309 | 96.69 | 1.65E-08 |
| NREP | neuronal regeneration related protein | 0.665487531 | -0.422316932 | 12.32 | 0.000013 |
| ZNF620 | zinc finger protein 620 | 0.659483037 | -0.464232067 | 46.42 | 0.002279838 |
| TNFAIP3 | TNF alpha induced protein 3 | 0.650378182 | -0.467341172 | 86.9 | 0.002472203 |
| NLRP3 | NLR family pyrin domain containing 3 | 0.629392919 | -0.623867534 | 113.96 | 0.00011852 |
| FBXL7 | F-box and leucine rich repeat protein 7 | 0.610131627 | -0.435390026 | 54.01 | 0.004159801 |
| RIPK4 | receptor interacting serine/threonine kinase 4 | 0.572334369 | -0.671162638 | 86.24 | 0.002888474 |
| ABCA1 | ATP binding cassette subfamily A member 1 | 0.568896568 | -0.670489466 | 248.71 | 0.001094561 |
| ABCA7 | ATP binding cassette subfamily A member 7 | 0.56136243 | -0.576246272 | 236.06 | 0.00277236 |
| SYTL4 | synaptotagmin like 4 | 0.557518578 | -0.765829972 | 73.81 | 0.001909398 |
| IL32 | interleukin 32 | 0.53651471 | -0.470897042 | 25.74 | 0.001250001 |
| CDKN1A | cyclin dependent kinase inhibitor 1A | 0.523233695 | -0.660138566 | 18.04 | 0.000114893 |
| CREG1 | cellular repressor of E1A stimulated genes 1 | 0.517025469 | -0.428246086 | 24.2 | 0.001228703 |
| HSPG2 | heparan sulfate proteoglycan 2 | 0.481283782 | -0.528178685 | 483.01 | 0.0000558 |
| JAG1 | jagged canonical Notch ligand 1 | 0.477371168 | -0.357132485 | 133.98 | 0.005769227 |
| ATP10D | ATPase phospholipid transporting 10D (putative) | 0.463162085 | -0.442709736 | 156.86 | 0.000513931 |
| FAM102A | estrogen-induced osteoclastogenesis regulator 1 | 0.444054863 | -0.335713559 | 42.24 | 0.003293779 |
| ARHGEF6 | Rac/Cdc42 guanine nucleotide exchange factor 6 | 0.435596825 | -0.581018134 | 85.36 | 0.006407456 |
| TGFB3 | transforming growth factor beta 3 | 0.43307013 | -0.487478549 | 45.32 | 0.007312125 |
| ITGA5 | integrin subunit alpha 5 | 0.419767325 | -0.398465152 | 115.39 | 0.002634053 |
| ITGA2 | integrin subunit alpha 2 | 0.399897465 | -0.537988235 | 129.91 | 0.002926241 |
| RAD9A | RAD9 checkpoint clamp component A | 0.385431037 | -0.350475721 | 43.01 | 0.001548014 |
| B4GALT5 | beta-1,4-galactosyltransferase 5 | 0.366964473 | -0.371462087 | 42.68 | 0.001951256 |
| BAHCC1 | BAH domain and coiled-coil containing 1 | 0.363118233 | -0.411251107 | 290.29 | 0.004339777 |
| AP5B1 | adaptor related protein complex 5 subunit beta 1 | 0.358142004 | -0.274214299 | 96.58 | 0.009149344 |
| UBN2 | ubinuclein 2 | 0.347639938 | -0.377291195 | 148.17 | 0.007572143 |
| RIPK1 | receptor interacting serine/threonine kinase 1 | 0.345749357 | -0.304136366 | 73.81 | 0.000394657 |
| DDR1 | discoidin domain receptor tyrosine kinase 1 | 0.343603218 | -0.329726477 | 101.09 | 0.000797182 |
| MICB | MHC class I polypeptide-related sequence B | 0.336559892 | -0.431648519 | 42.13 | 0.000815781 |
| IRAK4 | interleukin 1 receptor associated kinase 4 | 0.321811626 | -0.283940367 | 50.6 | 0.001188096 |
| SETX | senataxin | 0.295300806 | -0.266352302 | 294.47 | 0.00840714 |
| STX16 | syntaxin 16 | 0.285735289 | -0.291553584 | 35.75 | 0.006666357 |
| FRMD8 | FERM domain containing 8 | 0.284188756 | -0.644577361 | 51.04 | 0.006031623 |
| DYNLT1 | dynein light chain Tctex-type 1 | 0.27227859 | -0.293164619 | 12.43 | 0.004446773 |
| H2BC12 | H2B clustered histone 12 | 0.271014599 | -0.396520292 | 13.86 | 0.002532148 |
| HSDL2 | hydroxysteroid dehydrogenase like 2 | -0.273113654 | 0.423521916 | 45.98 | 0.007582664 |
| ZNF664 | zinc finger protein 664 | -0.274528691 | 0.508104386 | 28.71 | 0.005220531 |
| FARSB | phenylalanyl-tRNA synthetase subunit beta | -0.275513992 | 0.293796389 | 64.79 | 0.006122547 |
| TCF3 | transcription factor 3 | -0.286986153 | 0.490312565 | 71.94 | 0.002415184 |
| RABEPK | Rab9 effector protein with kelch motifs | -0.297338145 | 0.373585389 | 40.92 | 0.001600909 |
| RACK1 | receptor for activated C kinase 1 | -0.315568992 | 0.237073765 | 34.87 | 0.003958771 |
| EEF2 | eukaryotic translation elongation factor 2 | -0.342182855 | 0.330933786 | 94.38 | 0.001434347 |
| RPL13A | ribosomal protein L13a | -0.344448951 | 0.276020607 | 22.33 | 0.002231575 |
| NLE1 | notchless homolog 1 | -0.345163518 | 0.441853729 | 53.35 | 0.007860551 |
| LOC339803 | C2orf74 divergent transcript | -0.354006538 | 0.396993874 | NA | 0.004689125 |
| VOPP1 | VOPP1 WW domain binding protein | -0.355516639 | 0.329337495 | 18.92 | 0.000140442 |
| ATP5PB | ATP synthase peripheral stalk-membrane subunit b | -0.356486573 | 0.290980268 | 28.16 | 0.002335542 |
| ODC1 | ornithine decarboxylase 1 | -0.392387834 | 0.33492986 | 50.71 | 0.00644772 |
| PERP | p53 apoptosis effector related to PMP22 | -0.41114921 | 0.271614592 | 21.23 | 0.000439684 |
| IMPDH2 | inosine monophosphate dehydrogenase 2 | -0.423953595 | 0.31507976 | 56.54 | 0.000187276 |
| CDK14 | cyclin dependent kinase 14 | -0.425793339 | 0.483754798 | 51.59 | 0.002651788 |
| SETD7 | SET domain containing 7, histone lysine methyltransferase | -0.428650186 | 0.658888222 | 40.26 | 0.000242311 |
| FOPNL | centrosomal protein 20 | -0.449526394 | 0.315941915 | 11 | 0.000741039 |
| EIF3F | eukaryotic translation initiation factor 3 subunit F | -0.481711563 | 0.309275213 | 39.27 | 0.0000278 |
| FNBP1 | formin binding protein 1 | -0.504774038 | 0.448951142 | 67.87 | 0.00000113 |
| VAT1 | vesicle amine transport 1 | -0.512644611 | 0.307688129 | 43.23 | 0.001591124 |
