# Supplementary material for: FAM210B activates STAT1/IRF9/IFIT3 axis by upregulating IFN-α/β expression to impede the progression of lung adenocarcinoma
Source: Cell Death Dis. 2025 Feb 3;16(1):63. doi: 10.1038/s41419-025-07375-9 (PMC11791038; doi:10.1038/s41419-025-07375-9)
Supplement: Supplementary file 5 — Supplementary Table S4 [file 41419_2025_7375_MOESM5_ESM.docx]

**Table S4** The list of proteins interacting with FAM210B was identified using the IP-MS method.

| **Protein name** | **Description** | **Coverage [%]** | **Unique Peptides** | **# AAs** | **MW [kDa]** | **Protein FDR Confidence: Combined** |
| --- | --- | --- | --- | --- | --- | --- |
| FAM210B | Family with sequence similarity 210 member B | 65 | 14 | 192 | 20.4 | High |
| TOMM70 | Translocase of outer mitochondrial membrane 70 | 2 | 1 | 608 | 67.4 | High |
| FRYL | Protein furry homolog-like | 20 | 47 | 3013 | 339.4 | High |
| MON2 | Protein MON2 homolog | 31 | 44 | 1717 | 190.2 | High |
| NPEPPS | Puromycin-sensitive aminopeptidase | 47 | 38 | 919 | 103.2 | High |
| ATP2A2 | Sarcoplasmic/endoplasmic reticulum calcium ATPase 2 | 36 | 33 | 1042 | 114.7 | High |
| PPFIBP1 | Liprin-beta-1 | 34 | 27 | 1011 | 114 | High |
| XPOT | Exportin-T | 40 | 27 | 962 | 109.9 | High |
| MARS | Methionine--tRNA ligase, cytoplasmic | 36 | 25 | 900 | 101.1 | High |
| XPO1 | Exportin-1 | 29 | 25 | 1071 | 123.3 | High |
| ANXA2 | Annexin A2 | 76 | 24 | 339 | 38.6 | High |
| USP9X | Probable ubiquitin carboxyl-terminal hydrolase FAF-X | 10 | 23 | 2570 | 292.1 | High |
| IPO9 | Importin-9 | 29 | 22 | 1041 | 115.9 | High |
| SMC4 | Structural maintenance of chromosomes protein 4 | 19 | 22 | 1288 | 147.1 | High |
| WDR6 | WD repeat-containing protein 6 | 28 | 21 | 1121 | 121.6 | High |
| GEMIN4 | Gem-associated protein 4 | 25 | 21 | 1058 | 120 | High |
| CAND1 | Cullin-associated NEDD8-dissociated protein 1 | 20 | 20 | 1230 | 136.3 | High |
| VPS18 | Vacuolar protein sorting-associated protein 18 homolog | 28 | 20 | 973 | 110.1 | High |
| NUP205 | Nuclear pore complex protein Nup205 | 12 | 20 | 2012 | 227.8 | High |
| IPO5 | Importin-5 | 24 | 19 | 1097 | 123.6 | High |
| MCM7 | DNA replication licensing factor MCM7 | 34 | 19 | 719 | 81.3 | High |
| GEMIN5 | Gem-associated protein 5 | 16 | 19 | 1508 | 168.5 | High |
| TCP1 | T-complex protein 1 subunit alpha | 34 | 18 | 556 | 60.3 | High |
| SMC2 | Structural maintenance of chromosomes protein 2 | 18 | 18 | 1197 | 135.6 | High |
| GCN1 | eIF-2-alpha kinase activator GCN1 | 8 | 17 | 2671 | 292.6 | High |
| MCM3 | DNA replication licensing factor MCM3 | 23 | 17 | 808 | 90.9 | High |
| HCCS | Cytochrome c-type heme lyase | 52 | 16 | 268 | 30.6 | High |
| NUP188 | Nucleoporin NUP188 homolog | 12 | 16 | 1749 | 195.9 | High |
| IPO11 | Importin-11 | 19 | 16 | 975 | 112.5 | High |
| NUP160 | Nuclear pore complex protein Nup160 | 13 | 15 | 1436 | 162 | High |
| EIF2B3 | Translation initiation factor eIF-2B subunit gamma | 38 | 14 | 452 | 50.2 | High |
| EIF2B5 | Translation initiation factor eIF-2B subunit epsilon | 27 | 14 | 721 | 80.3 | High |
| EIF2B4 | Translation initiation factor eIF-2B subunit delta | 40 | 14 | 523 | 57.5 | High |
| DDX20 | Probable ATP-dependent RNA helicase DDX20 | 24 | 14 | 824 | 92.2 | High |
| CSE1L | Exportin-2 | 16 | 14 | 971 | 110.3 | High |
| RNF213 | E3 ubiquitin-protein ligase RNF213 | 4 | 14 | 5207 | 591 | High |
| EIF2B2 | Translation initiation factor eIF-2B subunit beta | 35 | 13 | 351 | 39 | High |
| PFAS | Phosphoribosylformylglycinamidine synthase | 12 | 13 | 1338 | 144.6 | High |
| LTN1 | E3 ubiquitin-protein ligase listerin | 10 | 13 | 1766 | 200.4 | High |
| TNPO3 | Transportin-3 | 21 | 13 | 923 | 104.1 | High |
| GBF1 | Golgi-specific brefeldin A-resistance guanine nucleotide exchange factor 1 | 7 | 13 | 1859 | 206.3 | High |
| TUBB6 | Tubulin beta-6 chain | 73 | 12 | 446 | 49.8 | High |
| EIF2B1 | Translation initiation factor eIF-2B subunit alpha | 48 | 12 | 305 | 33.7 | High |
| VPS41 | Vacuolar protein sorting-associated protein 41 homolog | 18 | 12 | 854 | 98.5 | High |
| XPO4 | Exportin-4 | 12 | 12 | 1151 | 130.1 | High |
| PPP2R1A | Serine/threonine-protein phosphatase 2A 65 kDa regulatory subunit A alpha isoform | 27 | 12 | 589 | 65.3 | High |
| DNAJA1 | DnaJ homolog subfamily A member 1 | 47 | 11 | 397 | 44.8 | High |
| NUP85 | Nuclear pore complex protein Nup85 | 23 | 11 | 656 | 75 | High |
| TNPO1 | Transportin-1 | 19 | 11 | 898 | 102.3 | High |
| ATP1A1 | Sodium/potassium-transporting ATPase subunit alpha-1 | 13 | 11 | 1023 | 112.8 | High |
| VPS33A | Vacuolar protein sorting-associated protein 33A | 23 | 11 | 596 | 67.6 | High |
| DNAJA2 | DnaJ homolog subfamily A member 2 | 28 | 11 | 412 | 45.7 | High |
| MMS19 | MMS19 nucleotide excision repair protein homolog | 11 | 11 | 1030 | 113.2 | High |
| VPS16 | Vacuolar protein sorting-associated protein 16 homolog | 18 | 10 | 839 | 94.6 | High |
| PPM1B | Protein phosphatase 1B | 31 | 10 | 479 | 52.6 | High |
| RPN1 | Dolichyl-diphosphooligosaccharide--protein glycosyltransferase subunit 1 | 24 | 10 | 607 | 68.5 | High |
| MDN1 | Midasin | 2 | 10 | 5596 | 632.4 | High |
| CCT8 | T-complex protein 1 subunit theta | 24 | 10 | 548 | 59.6 | High |
| SAMM50 | Sorting and assembly machinery component 50 homolog | 25 | 10 | 469 | 51.9 | High |
| NUP155 | Nuclear pore complex protein Nup155 | 9 | 9 | 1391 | 155.1 | High |
| ATP5F1B | ATP synthase subunit beta, mitochondrial | 21 | 9 | 529 | 56.5 | High |
| RANBP6 | Ran-binding protein 6 | 9 | 9 | 1105 | 124.6 | High |
| DDB1 | DNA damage-binding protein 1 | 12 | 9 | 1140 | 126.9 | High |
| PPP6R3 | Serine/threonine-protein phosphatase 6 regulatory subunit 3 | 13 | 9 | 873 | 97.6 | High |
| PCNA | Proliferating cell nuclear antigen OS=Homo sapiens | 41 | 9 | 261 | 28.8 | High |
| ACSL3 | Long-chain-fatty-acid--CoA ligase 3 OS=Homo sapiens | 14 | 9 | 720 | 80.4 | High |
| HUWE1 | E3 ubiquitin-protein ligase HUWE1 | 3 | 8 | 4374 | 481.6 | High |
| ZW10 | Centromere/kinetochore protein zw10 homolog | 16 | 8 | 779 | 88.8 | High |
| NUP107 | Nuclear pore complex protein Nup107 | 12 | 8 | 925 | 106.3 | High |
| IPO8 | Importin-8 | 10 | 8 | 1037 | 119.9 | High |
| MTHFD1 | C-1-tetrahydrofolate synthase, cytoplasmic | 8 | 8 | 935 | 101.5 | High |
| SAAL1 | Protein SAAL1 | 13 | 8 | 474 | 53.5 | High |
| PRKAA1 | 5'-AMP-activated protein kinase catalytic subunit alpha-1 | 17 | 8 | 559 | 64 | High |
| TUBB3 | Tubulin beta-3 chain | 62 | 7 | 450 | 50.4 | High |
| SPTLC1 | Serine palmitoyltransferase 1 | 19 | 7 | 473 | 52.7 | High |
| TBC1D15 | TBC1 domain family member 15 | 12 | 7 | 691 | 79.4 | High |
| BAG6 | Large proline-rich protein BAG6 | 11 | 7 | 1132 | 119.3 | High |
| PRMT9 | Protein arginine N-methyltransferase 9 | 9 | 7 | 845 | 94.4 | High |
| DNAJC7 | DnaJ homolog subfamily C member 7 | 17 | 7 | 494 | 56.4 | High |
| STRAP | Serine-threonine kinase receptor-associated protein | 22 | 7 | 350 | 38.4 | High |
| XPO5 | Exportin-5 | 8 | 7 | 1204 | 136.2 | High |
| ARFGEF3 | Brefeldin A-inhibited guanine nucleotide-exchange protein 3 | 4 | 7 | 2177 | 240.5 | High |
| NBAS | Neuroblastoma-amplified sequence | 3 | 7 | 2371 | 268.4 | High |
| ESYT1 | Extended synaptotagmin-1 | 8 | 6 | 1104 | 122.8 | High |
| THADA | Thyroid adenoma-associated protein | 4 | 6 | 1953 | 219.5 | High |
| PPP2R2A | Serine/threonine-protein phosphatase 2A 55 kDa regulatory subunit B alpha isoform | 18 | 6 | 447 | 51.7 | High |
| ALDH3A2 | Fatty aldehyde dehydrogenase | 16 | 6 | 485 | 54.8 | High |
| AARS | Alanine--tRNA ligase, cytoplasmic | 9 | 6 | 968 | 106.7 | High |
| CUL7 | Cullin-7 | 4 | 6 | 1698 | 191 | High |
| NCAPG2 | Condensin-2 complex subunit G2 | 6 | 6 | 1143 | 130.9 | High |
| SLC25A6 | ADP/ATP translocase 3 | 35 | 5 | 298 | 32.8 | High |
| HSP90B1 | Endoplasmin | 9 | 5 | 803 | 92.4 | High |
| CLPTM1 | Cleft lip and palate transmembrane protein 1 | 10 | 5 | 669 | 76 | High |
| NCAPD3 | Condensin-2 complex subunit D3 | 5 | 5 | 1498 | 168.8 | High |
| UGDH | UDP-glucose 6-dehydrogenase | 14 | 5 | 494 | 55 | High |
| PCBP2 | Poly(rC)-binding protein 2 | 32 | 5 | 365 | 38.6 | High |
| PPP4R3A | Serine/threonine-protein phosphatase 4 regulatory subunit 3A | 10 | 5 | 833 | 95.3 | High |
| OPA1 | Dynamin-like 120 kDa protein, mitochondrial | 7 | 5 | 960 | 111.6 | High |
| INF2 | Inverted formin-2 | 6 | 5 | 1249 | 135.5 | High |
| AIMP1 | Aminoacyl tRNA synthase complex-interacting multifunctional protein 1 | 25 | 5 | 312 | 34.3 | High |
| ACP1 | Low molecular weight phosphotyrosine protein phosphatase | 32 | 5 | 158 | 18 | High |
| ARMCX3 | Armadillo repeat-containing X-linked protein 3 | 18 | 5 | 379 | 42.5 | High |
| KPNA2 | Importin subunit alpha-1 | 11 | 5 | 529 | 57.8 | High |
| TBC1D2B | TBC1 domain family member 2B | 8 | 5 | 963 | 109.8 | High |
| SLC25A3 | Phosphate carrier protein, mitochondrial | 12 | 5 | 362 | 40.1 | High |
| OGA | Protein O-GlcNAcase | 7 | 5 | 916 | 102.8 | High |
| HK2 | Hexokinase-2 | 6 | 5 | 917 | 102.3 | High |
| DHPS | Deoxyhypusine synthase | 14 | 4 | 369 | 40.9 | High |
| YWHAG | 14-3-3 protein gamma | 34 | 4 | 247 | 28.3 | High |
| SMN1 | Survival motor neuron protein | 17 | 4 | 294 | 31.8 | High |
| YWHAE | 14-3-3 protein epsilon | 27 | 4 | 255 | 29.2 | High |
| CPSF3 | Cleavage and polyadenylation specificity factor subunit 3 | 8 | 4 | 684 | 77.4 | High |
| PPM1F | Protein phosphatase 1F | 13 | 4 | 454 | 49.8 | High |
| PHKA1 | Phosphorylase b kinase regulatory subunit alpha, skeletal muscle isoform | 6 | 4 | 1223 | 137.2 | High |
| CTPS1 | CTP synthase 1 | 8 | 4 | 591 | 66.6 | High |
| UQCRC2 | Cytochrome b-c1 complex subunit 2, mitochondrial | 11 | 3 | 453 | 48.4 | High |
| IGHG1 | Immunoglobulin heavy constant gamma 1 | 25 | 3 | 330 | 36.1 | High |
| ERLIN1 | Erlin-1 | 22 | 3 | 346 | 38.9 | High |
| ARFGEF1 | Brefeldin A-inhibited guanine nucleotide-exchange protein 1 | 3 | 3 | 1849 | 208.6 | High |
| RABGAP1 | Rab GTPase-activating protein 1 | 4 | 3 | 1069 | 121.7 | High |
| PCBP1 | Poly(rC)-binding protein 1 | 24 | 3 | 356 | 37.5 | High |
| USO1 | General vesicular transport factor p115 | 5 | 3 | 962 | 107.8 | High |
| TUBA1C | Tubulin alpha-1C chain | 71 | 2 | 449 | 49.9 | High |
| TUBB8 | Tubulin beta-8 chain | 25 | 2 | 444 | 49.7 | High |
| MTCH2 | Mitochondrial carrier homolog 2 | 10 | 2 | 303 | 33.3 | High |
| HLA-C | HLA class I histocompatibility antigen, Cw-6 alpha chain | 15 | 2 | 366 | 40.9 | High |
| SCD | Acyl-CoA desaturase | 11 | 2 | 359 | 41.5 | High |
| ERLIN2 | Erlin-2 | 18 | 2 | 339 | 37.8 | High |
| SLC25A13 | Calcium-binding mitochondrial carrier protein Aralar2 | 8 | 2 | 675 | 74.1 | High |
| VDAC3 | Voltage-dependent anion-selective channel protein 3 | 11 | 2 | 283 | 30.6 | High |
| USP5 | Ubiquitin carboxyl-terminal hydrolase 5 | 4 | 2 | 858 | 95.7 | High |
| STOML2 | Stomatin-like protein 2, mitochondrial | 8 | 2 | 356 | 38.5 | High |
| TARDBP | TAR DNA-binding protein 43 | 3 | 1 | 414 | 44.7 | High |
| TBC1D5 | TBC1 domain family member 5 | 2 | 1 | 795 | 88.9 | High |
| PPIL1 | Peptidyl-prolyl cis-trans isomerase-like 1 | 10 | 1 | 166 | 18.2 | High |
| CKAP4 | Cytoskeleton-associated protein 4 | 3 | 1 | 602 | 66 | High |
| PSMA4 | Proteasome subunit alpha type-4 | 6 | 1 | 261 | 29.5 | High |
| TPM4 | Tropomyosin alpha-4 chain | 6 | 1 | 248 | 28.5 | High |
