# Supplementary material for: FAM210B activates STAT1/IRF9/IFIT3 axis by upregulating IFN-α/β expression to impede the progression of lung adenocarcinoma
Source: Cell Death Dis. 2025 Feb 3;16(1):63. doi: 10.1038/s41419-025-07375-9 (PMC11791038; doi:10.1038/s41419-025-07375-9)
Supplement: Supplementary file 6 — Original data [file 41419_2025_7375_MOESM6_ESM.pdf]

The raw images of western blots

Full and uncropped western blot for Figure 1E

Lanes 1, 2, 3 are on the figure

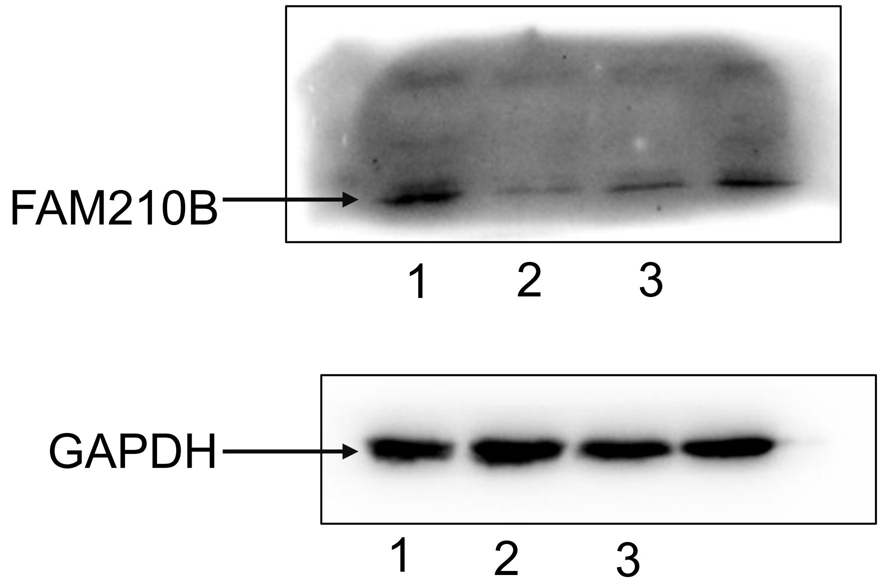

Full and uncropped western blot for Figure 2A

Lanes 1, 2 are on the figure

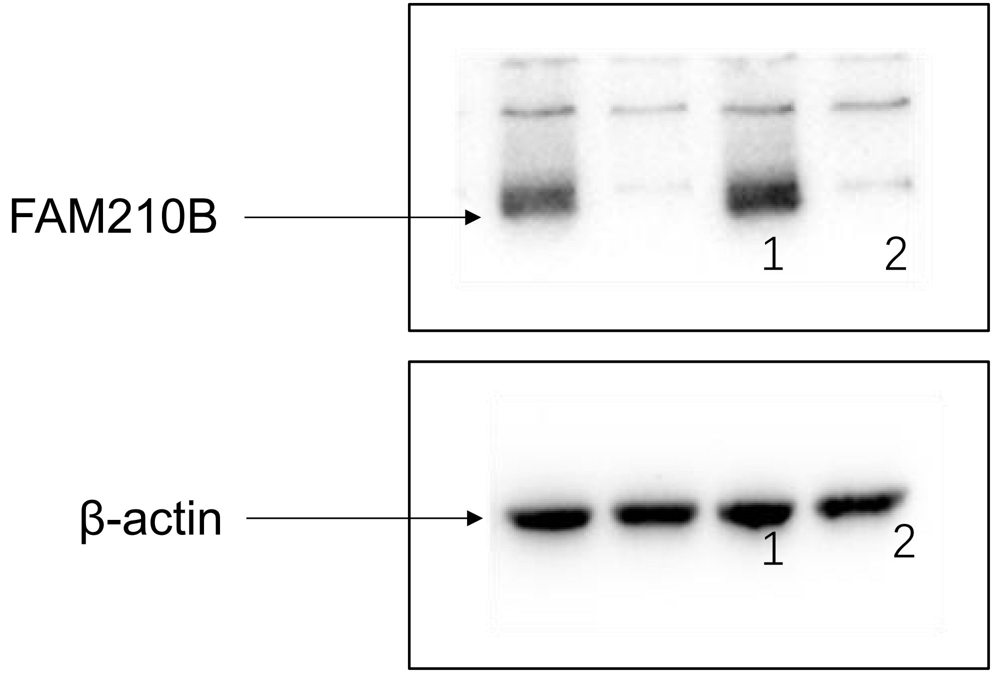

Full and uncropped western blot for Figure 2D

Lanes 1, 2 are on the figure

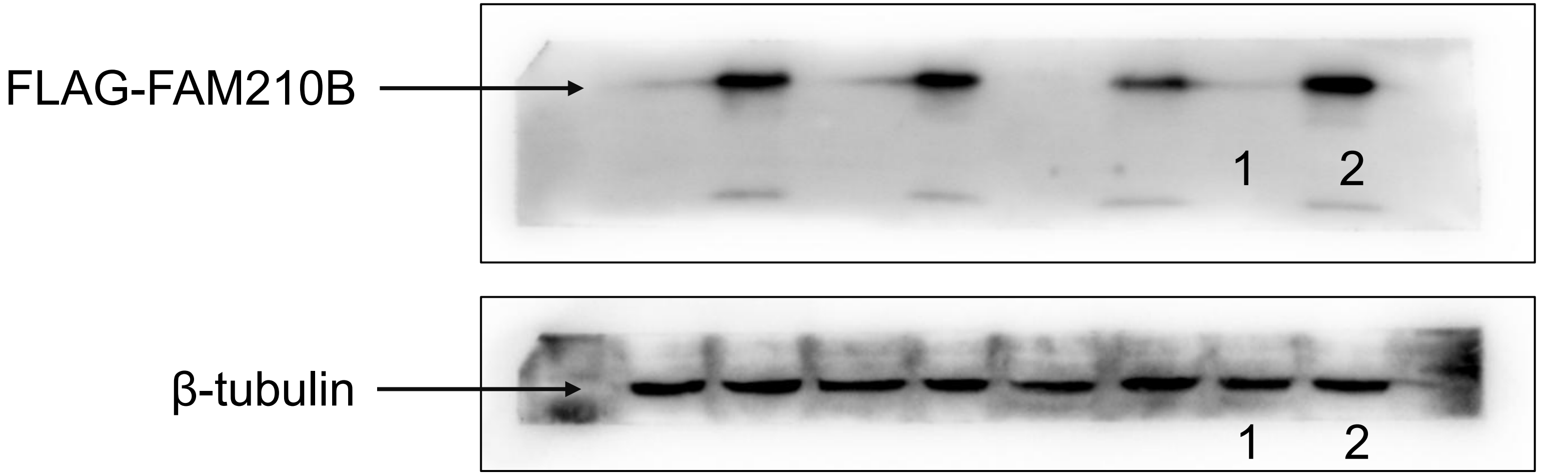

Full and uncropped western blot for Figure 2E

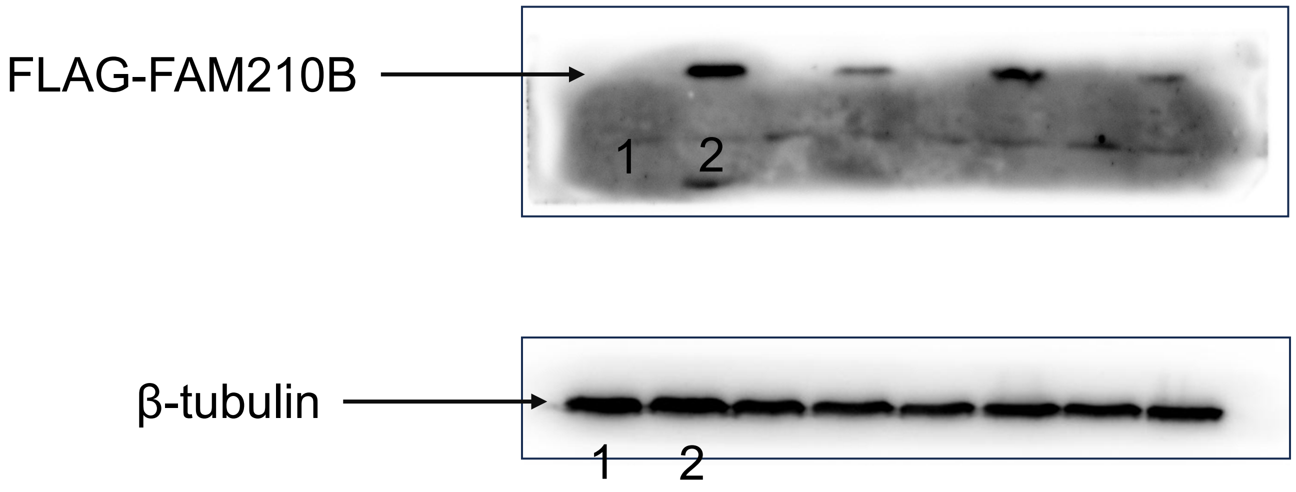

Full and uncropped western blot for Figure 2I

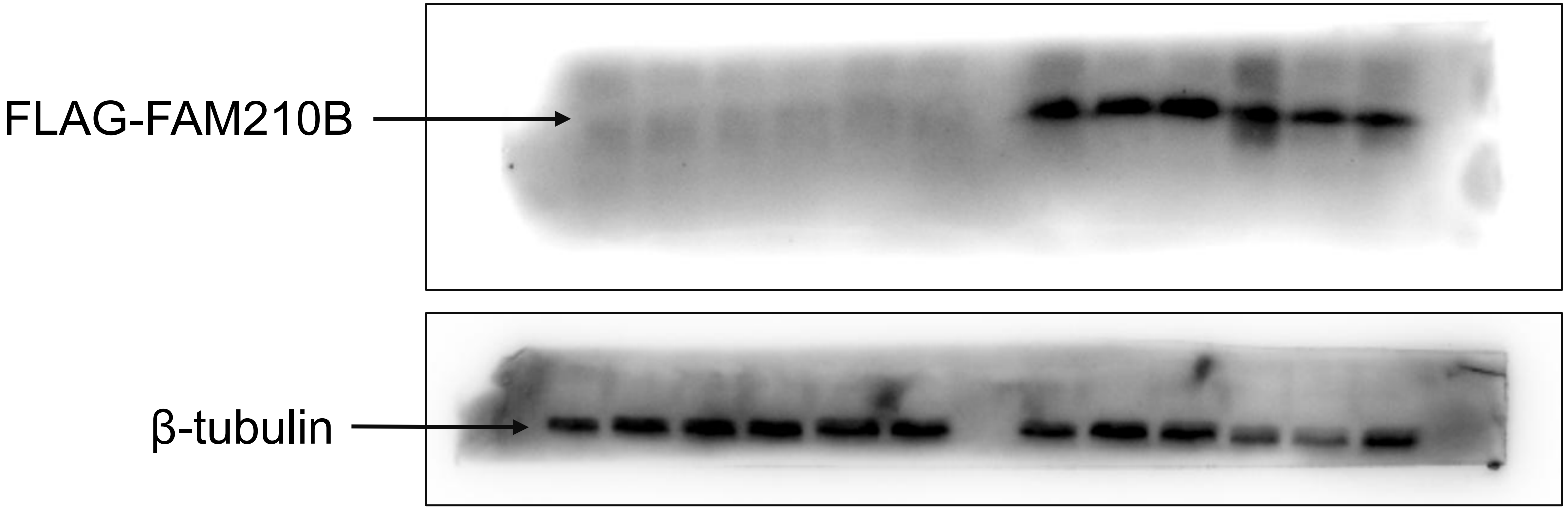

Full and uncropped western blot for Figure 3D left panel

Lanes 1, 2 are on the figure

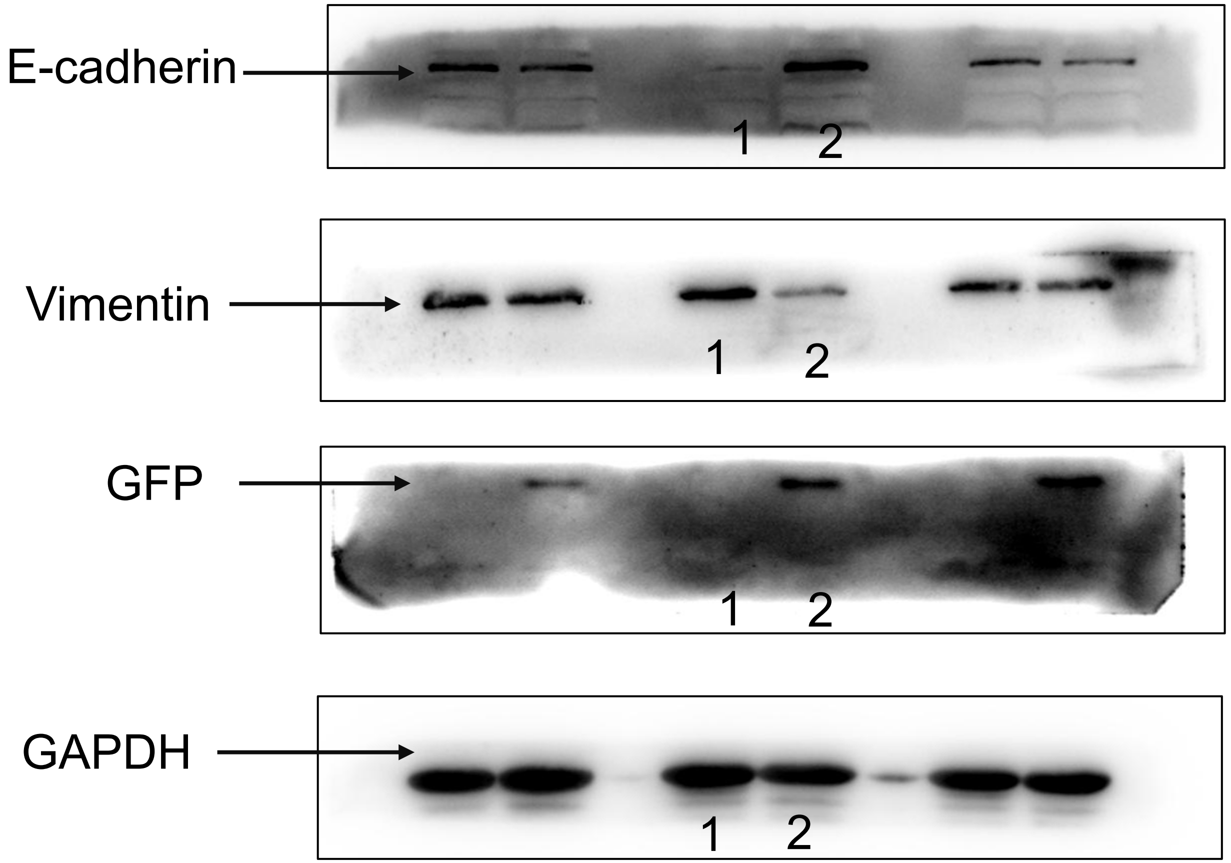

Full and uncropped western blot for Figure 3D right panel

Lanes 1, 2 are on the figure

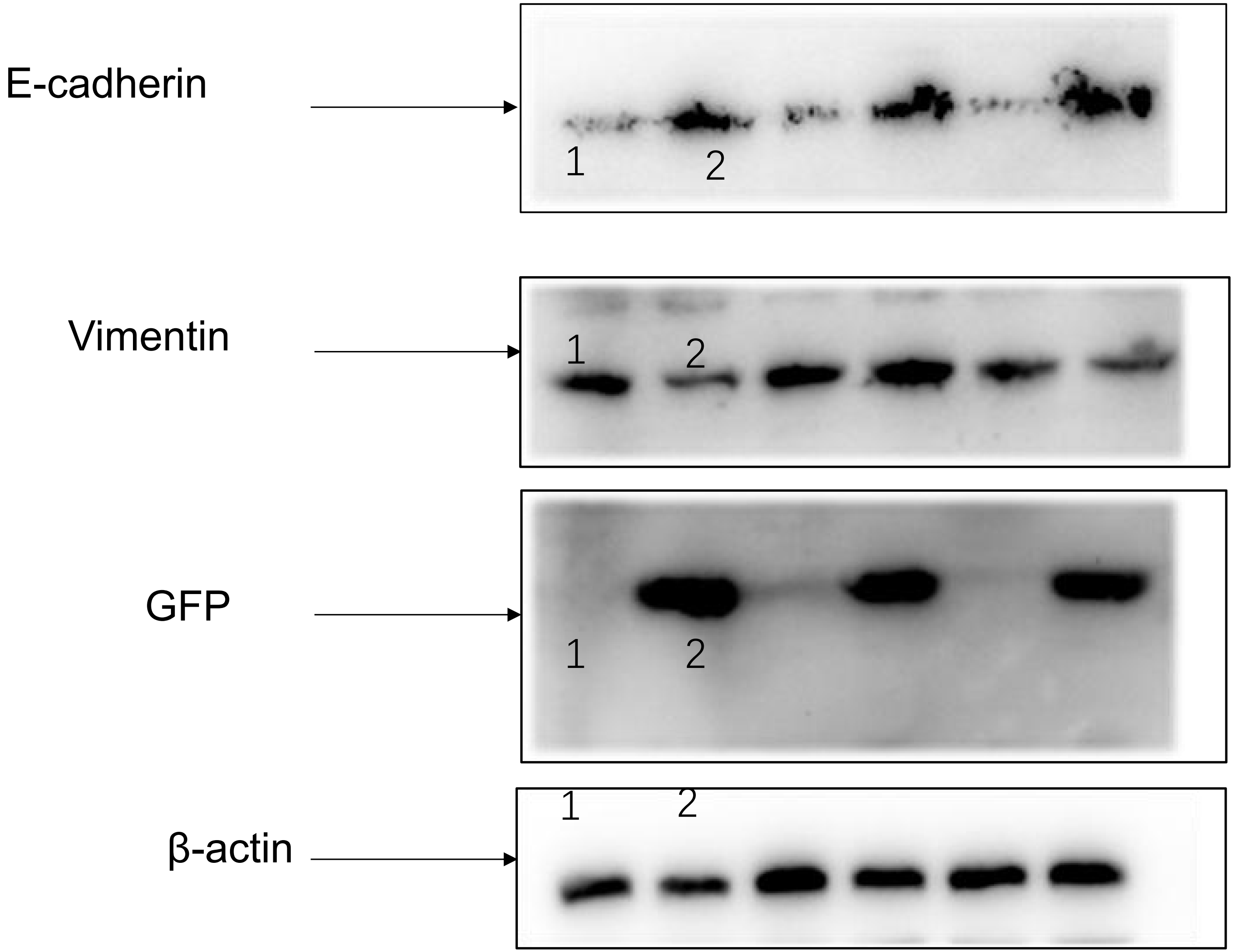

Full and uncropped western blot for Figure 3E

Lanes 1, 2 are on the figure

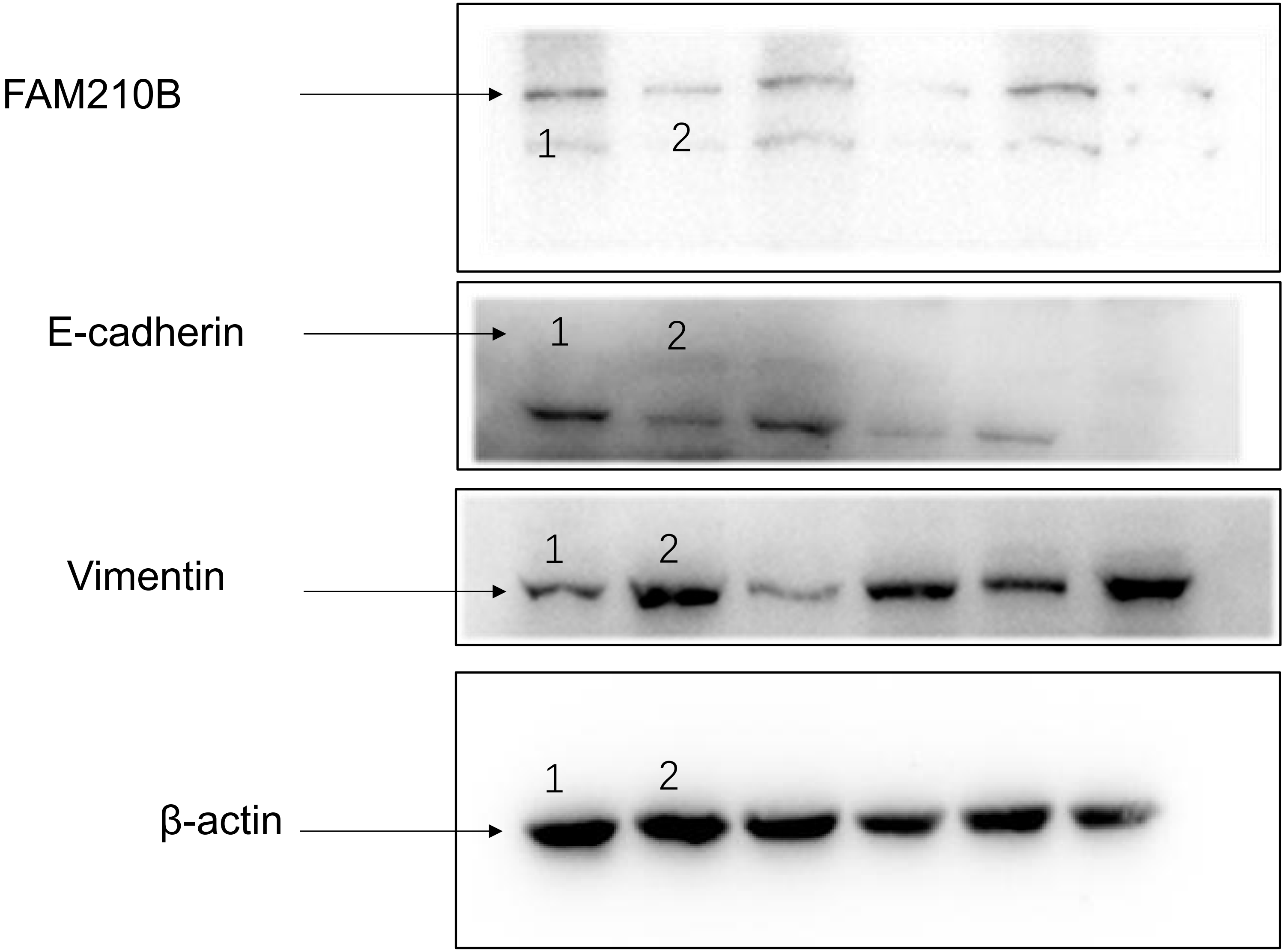

Full and uncropped western blot for Figure 4F left panel

Lanes 1, 2 are on the figure

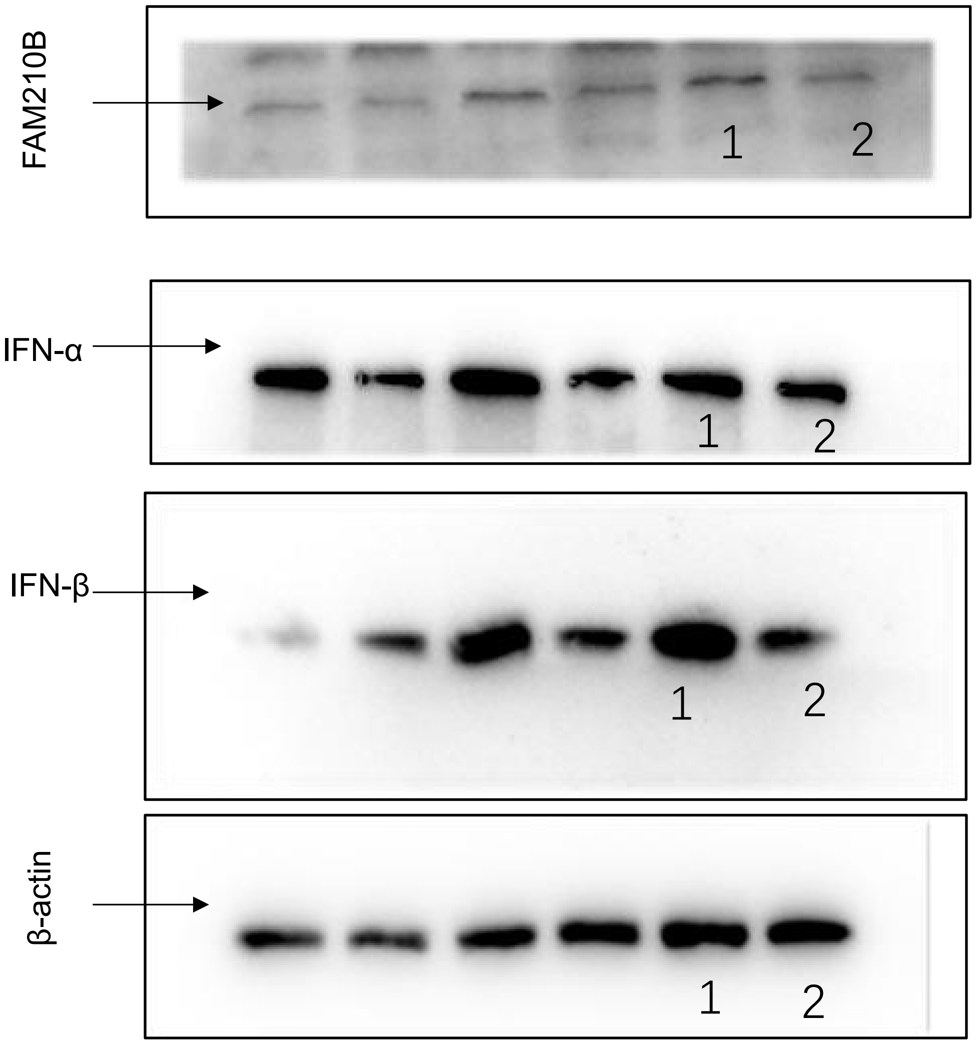

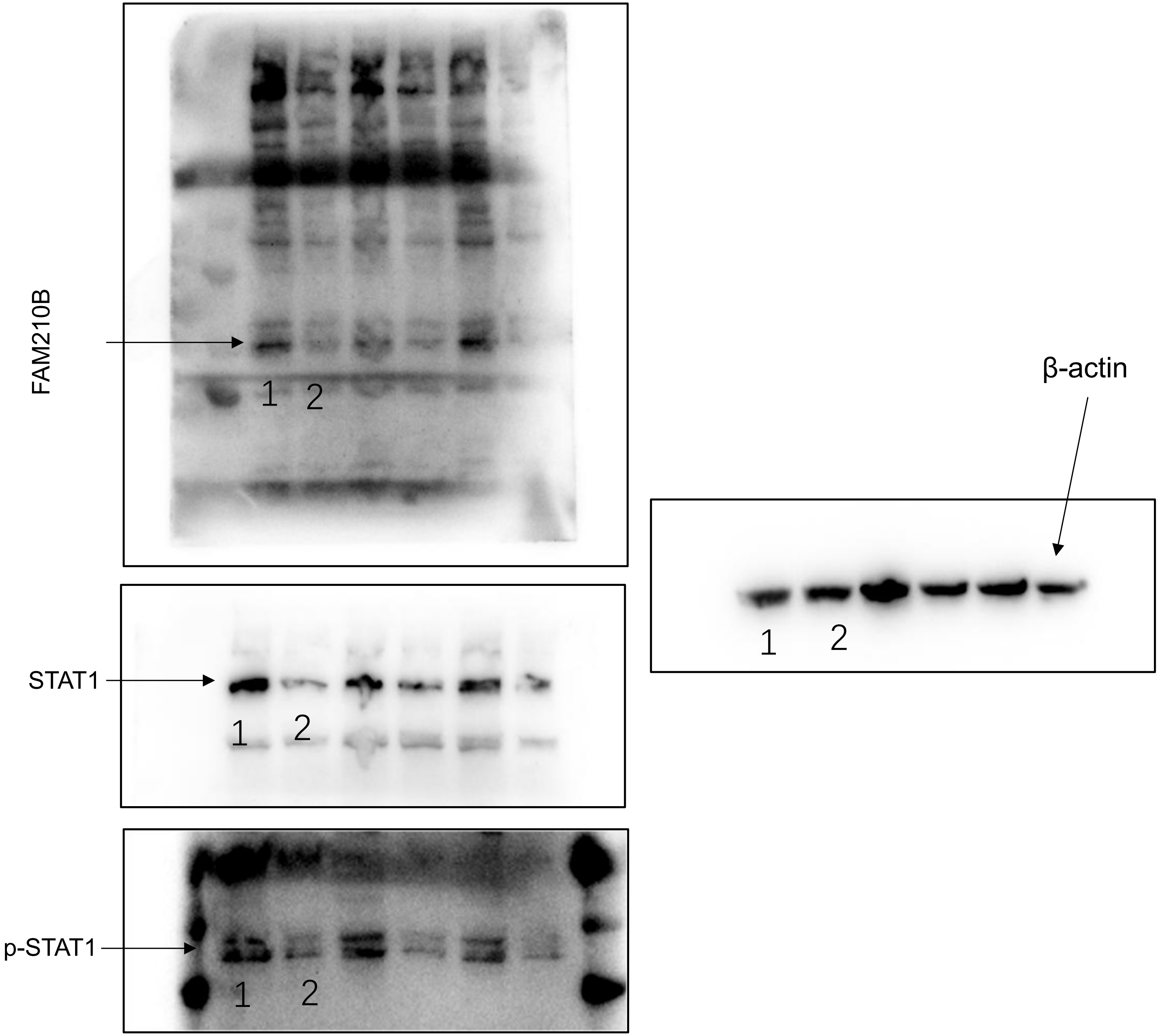

Full and uncropped western blot for Figure 4F middle panel

Lanes 1, 2 are on the figure

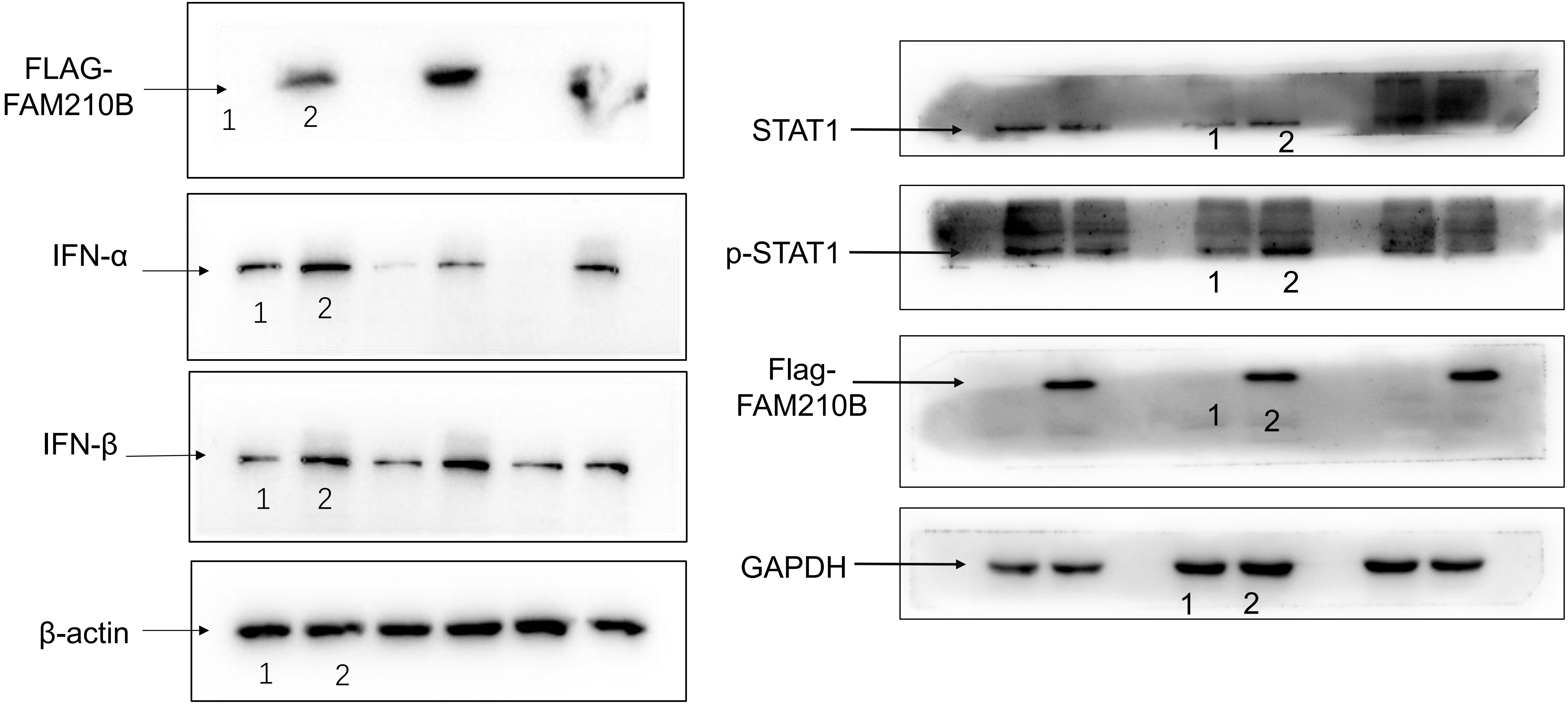

Full and uncropped western blot for Figure 4F right panel

Lanes 1, 2 are on the figure

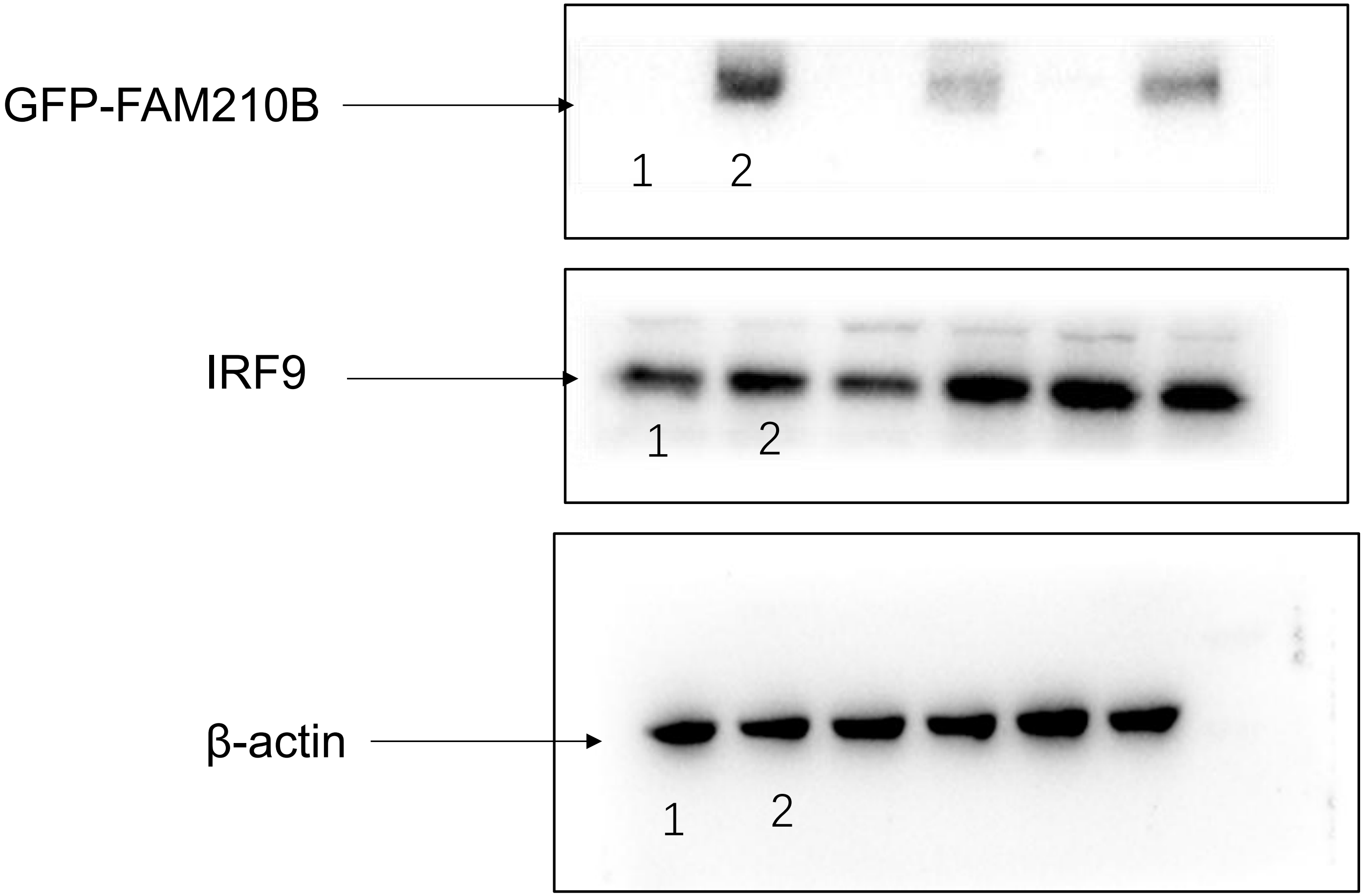

Full and uncropped western blot for Figure 6A

Lanes 1, 2 are on the figure

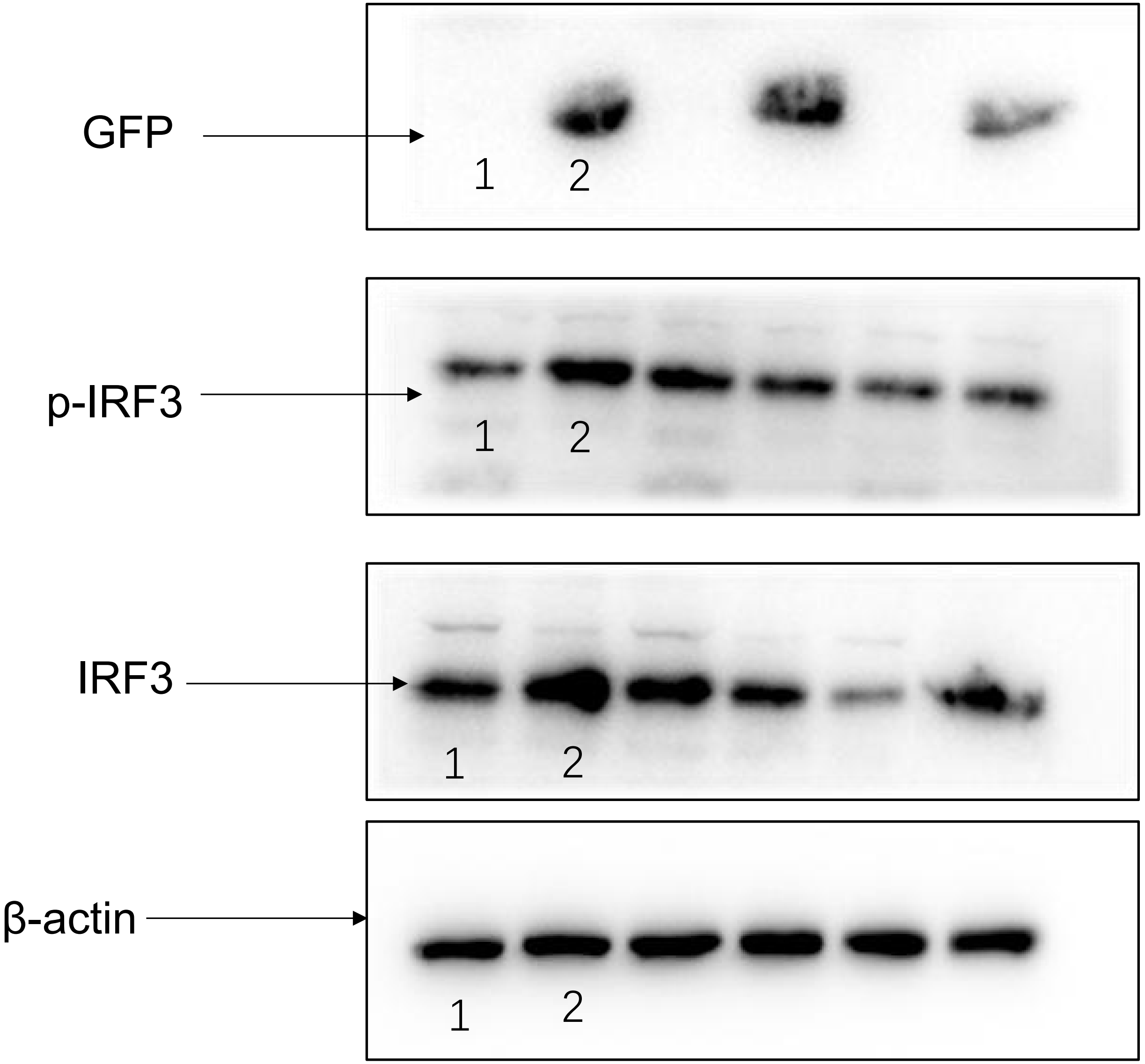

Full and uncropped western blot for Figure 6B

Lanes 1, 2,3,4 are on the figure

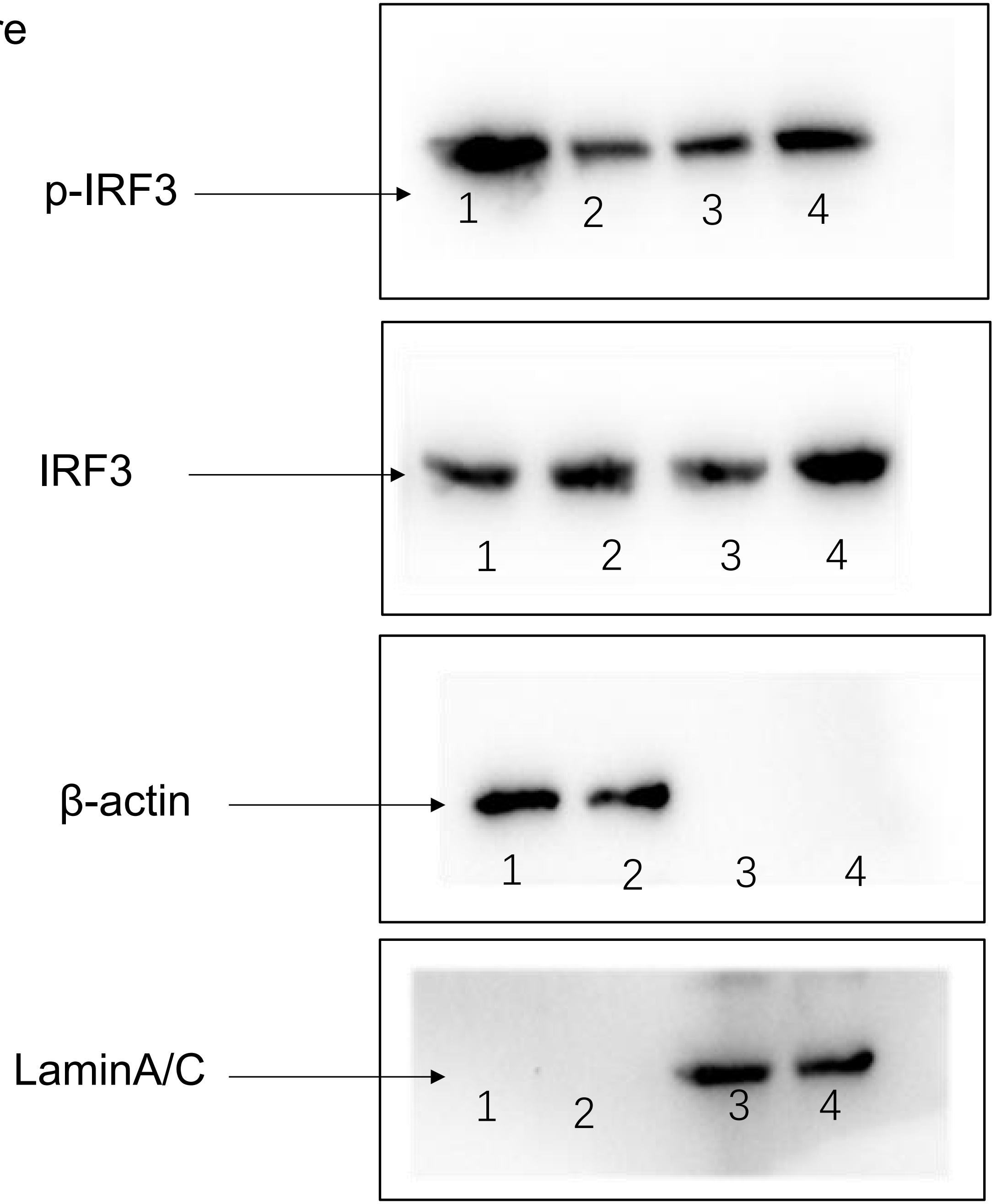

Full and uncropped western blot for Figure 6E

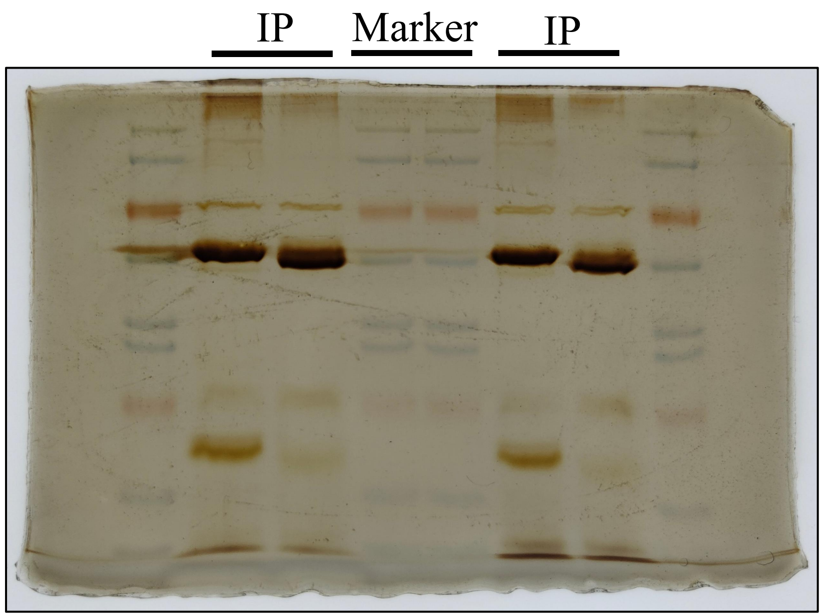

Full and uncropped western blot for Figure 6F

Lanes 1, 2, 3 are on the figure

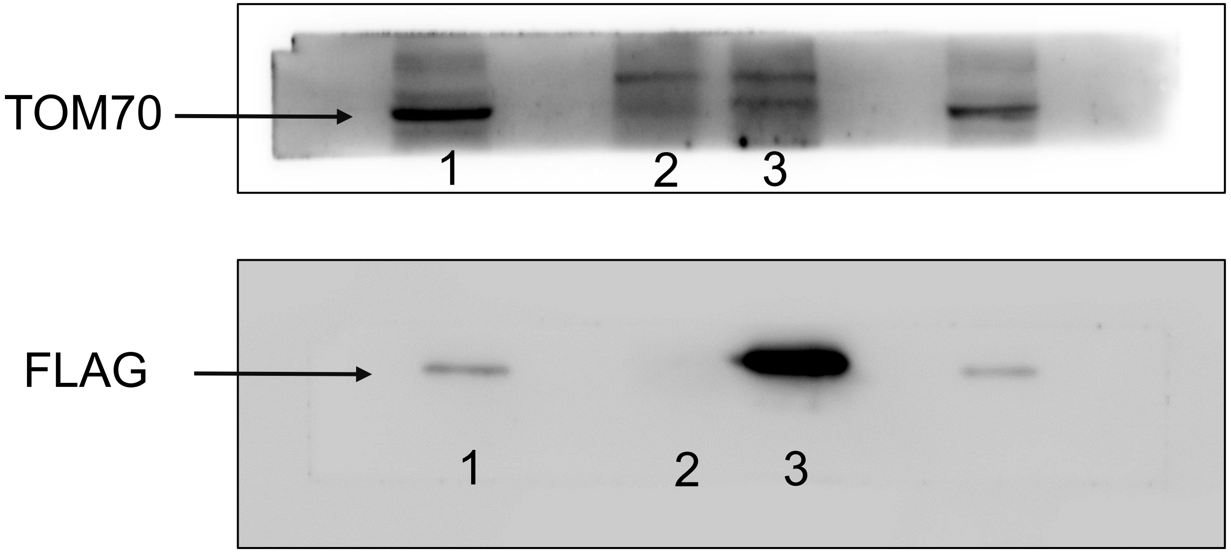

Full and uncropped western blot for Figure S2D

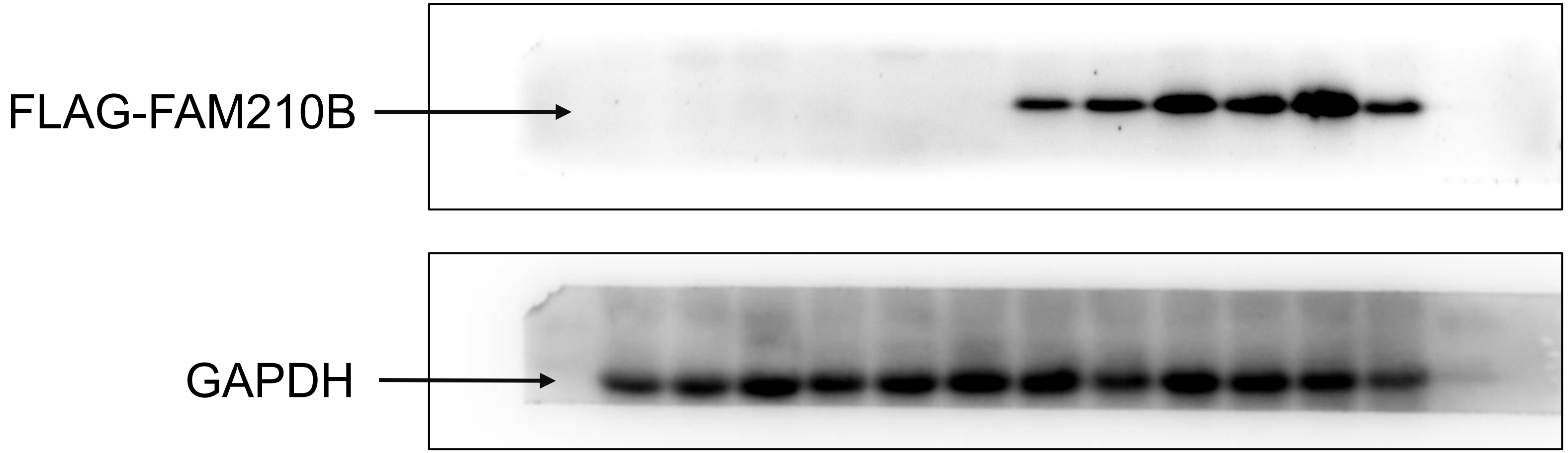

Full and uncropped western blot for Figure S3A

Lanes 1, 2,3,4 are on the figure

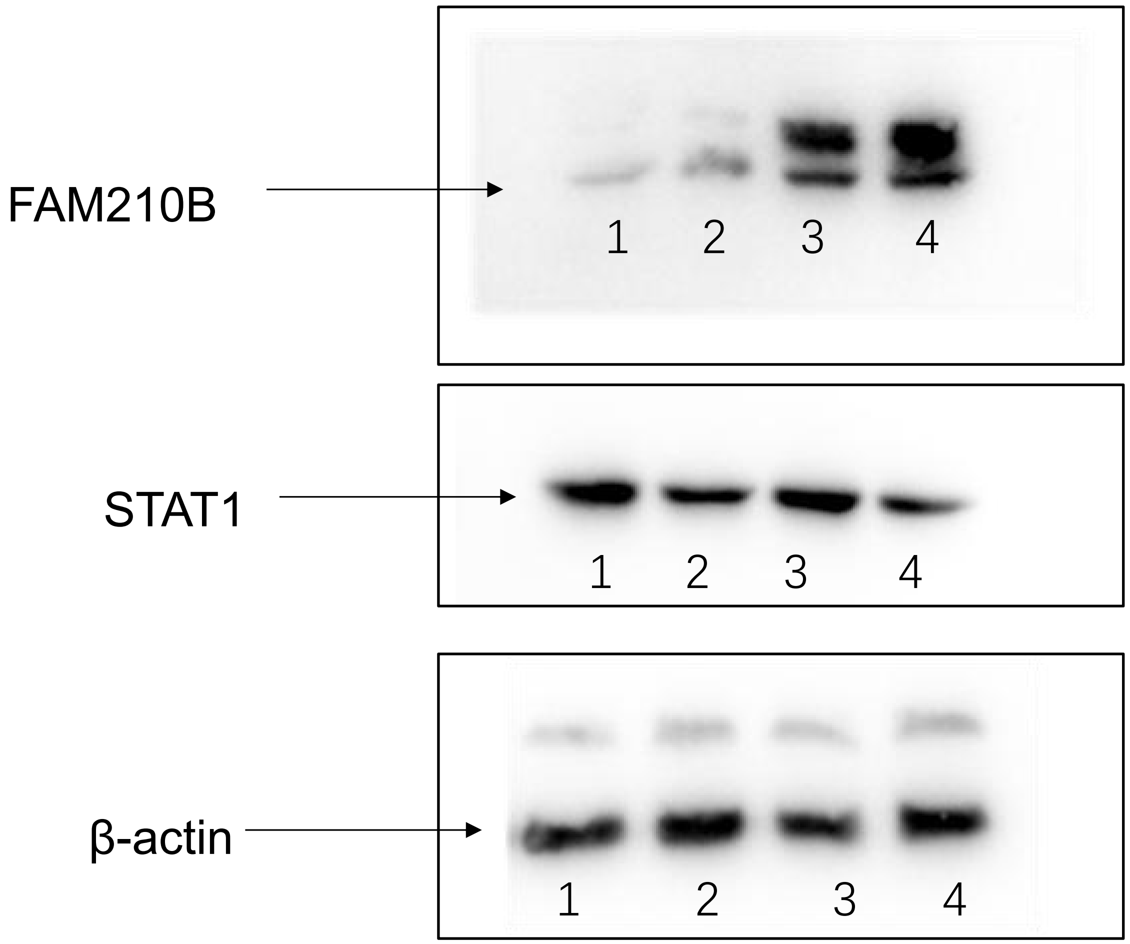

Full and uncropped western blot for Figure S3B

Lanes 1, 2,3,4 are on the figure

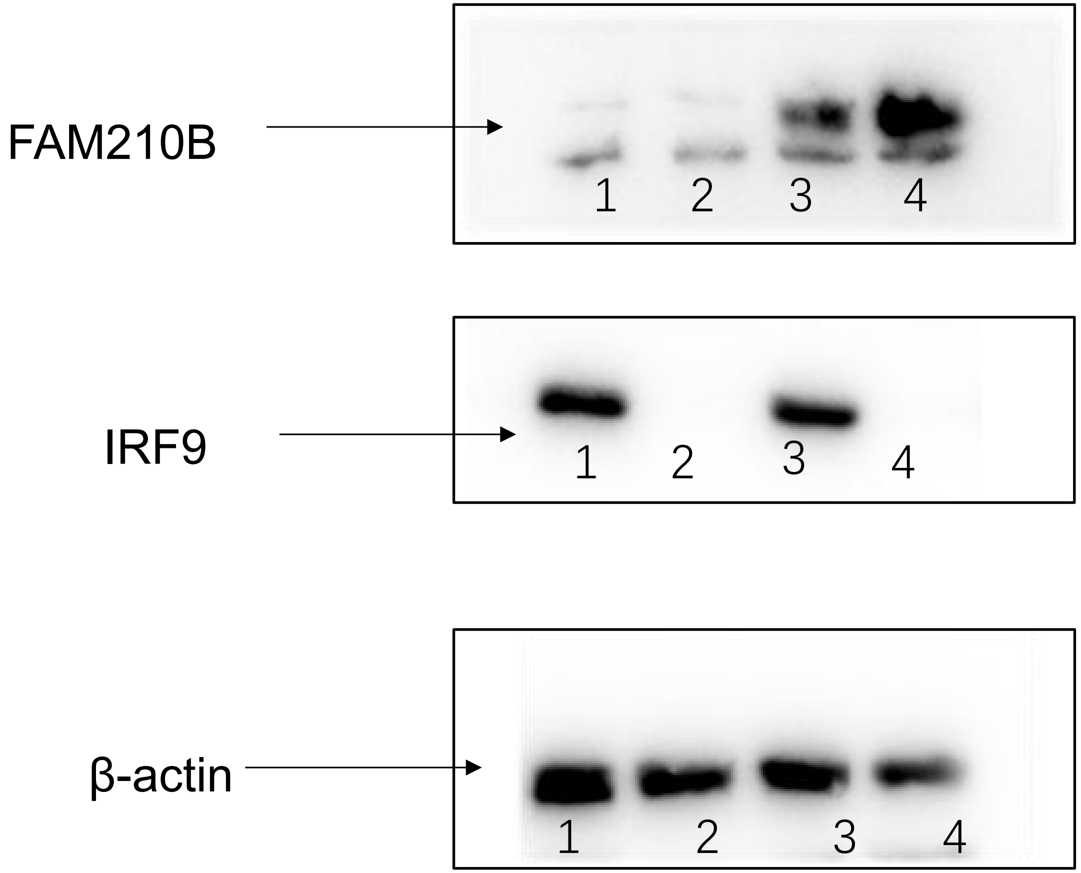

Full and uncropped western blot for Figure S3C

Lanes 1, 2,3,4 are on the figure

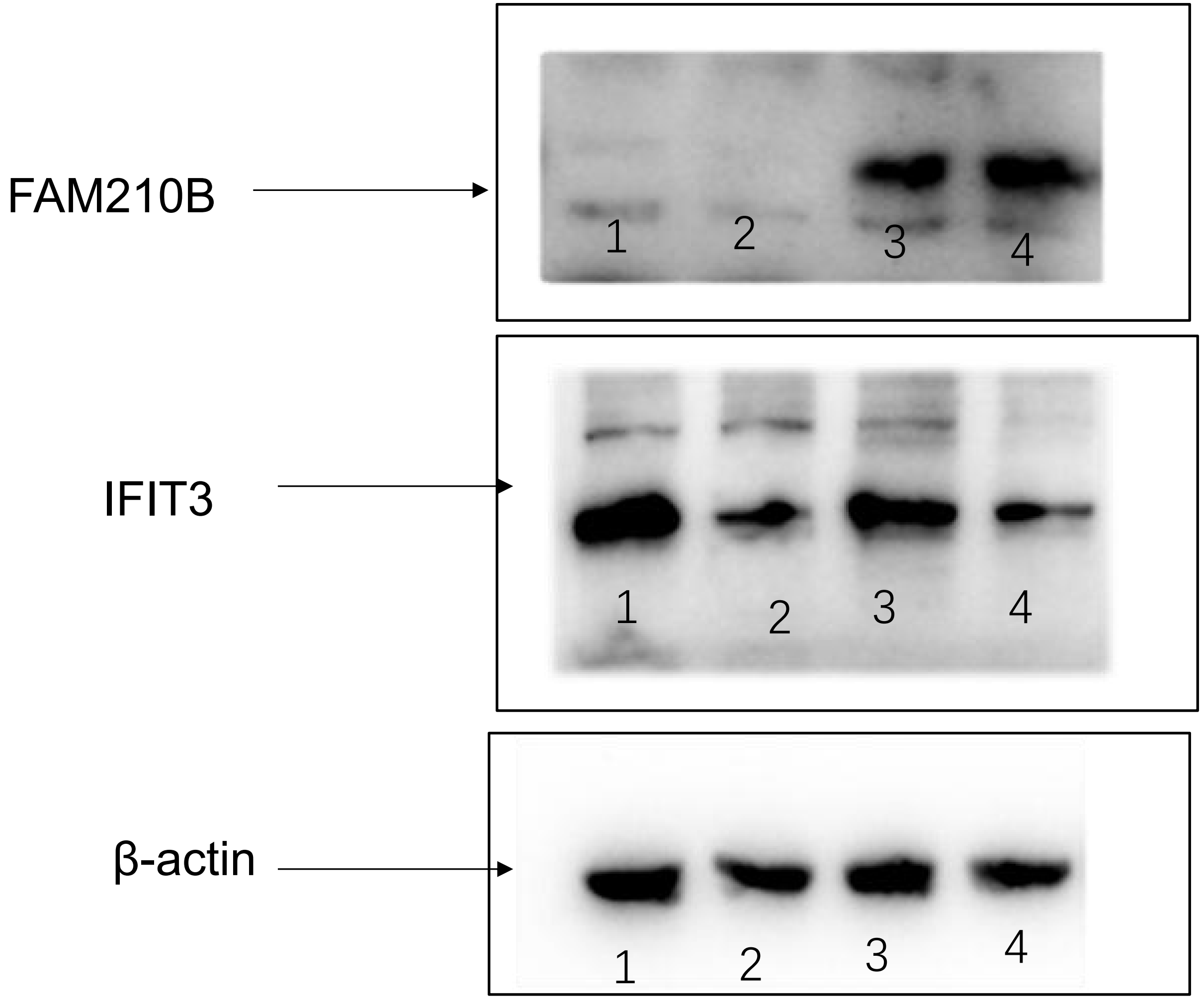

Original qRT-PCR data

Fig. 1D	HBE				H1299				A549			
FAM210B	1	1	0.9999	0.999999	0.3160871	0.2010508	0.2518669	0.4126942	0.5370027	0.3463602	0.3183036	0.2252771

Fig. 4D	GFP-V			GFP-FAM210B		
FAM210B	1	1	0.999999	18171.53	6585.581	12308.79
IFN-α	1	1	0.999999	2.414319	2.28470166<br>7	3.756828
IFN-β	1	1	0.999999	9.59774	7.508286	11.73926
STAT1	1	1	0.999999	2.514318	2.515088	2.738376
IRF9	1	1	0.999999	2.322228	1.681695	2.06789
IFIT3	1	1	0.999999	1.329806	1.73332	1.614356
IFIT1	1	1	0.999999	2.418706	1.995789	2.110597

Fig.4E	si-NC			si-FAM210B		
FAM210B	1	1	0.999999	0.5260793	0.2825003	0.1144623
IRF9	1	1	0.999999	0.6797261	0.682541	0.558054
IFIT3	1	1	0.999999	0.5775238	0.3042695	0.5267525
IFIT1	1	1	0.999999	0.6505828	0.5189674	0.7692931
FAM210B	1	1	1	0.2145224	0.3514254	0.374515
IFN-α	1	1	1	0.3569872	0.5236514	0.468214
IFN-β	1	1	1	0.458831	0.5422526	0.3352471
STAT1	1	1	1	0.5982457	0.442635	0.6414272

Fig. 4G	STAT1		IRF9		IFIT3	
0h	1	1	1	1	1	1
2h	2.281894	3.906674	5.05877	5.628143	5.434026	7.847174
4h	4.03864	5.499567	6.608851	8.035857	24.61707	36.06692
8h	6.920779	10.31893	7.334646	14.19596	45.84089	78.86096
12h	6.903573	9.473311	5.188531	6.99504	24.02033	29.2328

Fig. 4H	STAT1		IRF9		IFIT3	
0h	1	1	1	1	1	1
2h	2.502904	3.376156	5.816	4.592522	31.28483	32.43657
4h	4.476322	4.834388	10.88778	7.66865	90.5793	79.45606
8h	9.473878	9.909095	14.39938	10.28673	212.9488	196.7545
12h	7.645918	8.865585	15.05613	9.777695	149.9887	179.2751

Fig. 6D	OE-Vector+si-NC			OE-Vector+si-IRF3			OE-FAM210B+si-NC			OE-FAM210B+si-IRF3		
FAM210B	1	1	1	0.5735213	0.8272356	0.693654	99.78349	72.65424	110.2587	35.72968	55.35241	71.65824
IRF3	1	1	1	0.1944118	0.3268541	0.4023213	1.459737	1.908512	1.495423	0.1442399	0.552362	0.7932154
IFN-α	1	1	1	0.5520286	0.6854162	0.423851	8.271956	3.519854	4.923452	0.6730614	1.25655	1.304254
IFN-β	1	1	1	0.3105969	0.6325842	0.253654	20.51882	13.35412	12.2531	1.128939	0.254372	2.365427

Fig. 6K	OE-Vectoe+si-NC			OE-Vectoe+si-TOM70			OE-FAM210B+si-NC			OE-FAM210B+si-TOM70		
FAM210B	1	1	1	1.037955	1.147953	0.9313788	124.1816	129.9406	201.1879	24.36336	28.27543	14.90578
TOM70	1	1	1	0.1731674	0.2306067	0.3657171	0.8427804	0.9188662	0.922983	0.1723059	0.1997186	0.392223
IFN-α	1	1	1	0.1690381	0.5048604	0.534213	2.357015	1.77677	2.192444	0.6352838	0.6944962	0.604058
IFN-β	1	1	1	0.2640757	0.605066	0.1710156	7.17304	5.880976	3.798421	0.3750218	0.3935934	0.592086

Fig. S4A	si-NC			si-STAT1		
STAT1	1	1	1	0.3316127	0.3310356	0.3758662
FAM210B	1	1	1	0.6838511	0.7893187	0.6076997
TOM70	1	1	1	1.133792	1.013544	1.095502

Fig. S4B	si-NC			si-IRF9		
IRF9	1	1	1	0.3913071	0.5577567	0.4167466
FAM210B	1	1	1	0.5641417	0.6407855	0.7039922
TOM70	1	1	1	0.8022901	0.7316277	0.7778697

Fig. S4C	si-NC			si-IRF3		
IRF3	1	1	1	0.6090195	0.5847792	0.641323
FAM210B	1	1	1	0.8641417	0.7878547	0.8235392
TOM70	1	1	1	0.9689875	1.134852	1.099601

Fig. S5	OE-Vector				OE-FAM210B			
IFN-α	1	1	1	1	4.186399	5.898855	4.204623	3.290153
IFN-β	1	1	1	1	3.815828	5.151497	4.837729	3.611261

Fig. S6A	GFP-V			GFP-FAM210B		
FAM210B	1	1	1	3490.515	6568.542	3419.557
MDA-5	1	1	1	2.004368	2.087792	2.427388
RIG-1	1	1	1	1.949772	1.906862	1.535688

Fig. S6B	OE-V			OE-FAM210B		
FAM210B	1	1	0.999999	103.143	45.56583	92.68666
MDA5	1	1	0.999999	2.209857	2.136699	2.689101
RIG-I	1	1	0.999999	1.96135	2.126448	5.321416

Fig. S6C	0H			2H			4H		
MDA5	1	1	1	13.33176	12.65	14.06	25.91136	27.26	24.65
	6H			8H			12H		
MDA5	63.66199	69.25	58.57	141.3693	151.49	132.02	268.6505	270.48	267.04
Fig. S6C	0H			2H			4H		
RIG-I	1	1	1	1.575467	1.72	1.44	2.735077	3.15	2.37
	6H			8H			12H		
RIG-I	4.155257	4.75	3.63	7.326462	8.37	6.42	14.11163	15.63	12.74

Fig. S6D	0H			2H			6H		
MDA5	1	1	1	4.741819	4.441138	5.062859	4.624478	3.962361	5.397236
	8H			12H					
MDA5	13.10545	10.35696	16.58333	21.42684	21.39365	21.46009			

Fig. S6D	0H			2H			6H		
RIG-I	1	1	1	1.136389	1.233555	2.778223	1.513431	1.638372	1.398018
	8H			12H					
RIG-I	4.158895	3.999113	4.325062	6.59499	7.754469	5.608881			

Fig. S6E	OE-Vector+si-NC			OE-Vector+si-MDA5			OE-FAM210B+si-NC			OE-FAM210B+si-MDA5		
MDA5	1	1	1	0.116411	0.4374266	0.043009	1.699888	1.55728	1.097423	0.144639	0.646297	0.430573
IFN-α	1	1	1	0.420947	0.4845277	0.451938	7.583212	4.360695	3.39784	1.51656	2.02423	0.36712
IFN-β	1	1	1	0.281554	0.3319389	0.338747	11.19156	5.24543	8.921374	1.556817	0.458503	0.88317
